# Gene Expression and Alternative Splicing Throughout the Reproductive Cycle of a Viviparous Lizard Reveal Novel Genes for Pregnancy and Convergence With Squamates and Mammals

**DOI:** 10.1111/mec.70469

**Published:** 2026-07-21

**Authors:** John L. Smout, Maureen M. Bain, Mark McLaughlin, Kathryn R. Elmer

**Affiliations:** ^1^ School of Biodiversity, One Health and Veterinary Medicine, College of Medical, Veterinary & Life Sciences University of Glasgow Glasgow UK

## Abstract

Squamate pregnancy is an important topic in reproductive and evolutionary biology, as it provides insight to amniote live‐bearing that is not mammalian. Gene regulation in squamates is poorly characterised and the extent of molecular convergence in the developmental mechanisms of pregnancy across amniotes is strongly debated. To test the role of gene expression and alternative splicing in viviparous pregnancy in a lizard, we sequenced the uterine transcriptome of viviparous female Eurasian common lizards (Lacertidae: *Zootoca vivipara*) using long reads and examined whole‐gene and transcript‐level expression before, during, and after pregnancy. We found that 874 genes were differentially expressed between pregnant and non‐pregnant uterine tissue, including several involved in pregnancy in other animals, such as NOS1, MALL, and CLCN2. Upregulated genes were enriched for functional terms relevant to pregnancy in other amniotes, such as cardiovascular, tissue remodelling, and muscle‐related processes; downregulated terms were dominated by ribosomal activity. We found differential transcript usage between reproductive stages for 114 genes, of which most (78 genes) were not differentially expressed, suggesting alternative splicing and expression play different functional roles. Genes showing differential transcript usage included several with isoform variants involved in pregnancy in mammals and/or squamates, such as SRSF2, FAU, and LPP. These candidates involved alternative promoters and complex patterns of regulation varying by isoform across reproductive stages. Our study provides high‐resolution information about gene regulation during viviparous lizard reproduction, indicating a functional role of differential expression and alternative splicing involving new candidates and others known to be involved in amniote pregnancy.

## 1 Introduction

The birth of offspring is a fundamental aspect of animal biology, differing in its evolutionary history across the animal kingdom but convergent in function (Van Dyke et al. 2014; Whittington et al. 2025). Viviparous pregnancy is the full development of one or more offspring wholly within the maternal reproductive tract, the physiological process which defines viviparity. In amniotes, viviparous pregnancy developed from ancestral oviparity, in which animals deposit their young in hard‐shelled eggs that complete embryonic development outside the mother's body. Oviparity is seen in all extant non‐squamate sauropsids (birds, crocodilians, testudines, and Tuataras), a handful of mammals comprising the order Monotremata, and the majority of squamate reptiles (Blackburn 2006).

Pregnancy involves significant changes to the structure and function of the reproductive tract including extended retention of the developing embryo (Andrews 1997; Shine and Guillette 1988), an increase in the supply of gases, water and nutrients to the growing embryo in utero through augmented vascularisation and increasingly specialised placental structures (Linville et al. 2010; Mathies and Andrews 1996), elimination of the eggshell (Guillette 1993), and modulation of the maternal immune response to the developing embryo (Graham et al. 2011; Saad and El Deeb 1990). In matrotrophic pregnancy, seen in mammals and some derived viviparous squamates, pregnancy entails the delivery of nutrients to the growing embryo from the mother's bloodstream via the placenta (Blackburn 1992; Blackburn and Flemming 2010). In most viviparous squamates, however, the embryo still depends on the yolk‐sac for all its nutritional requirements, termed lecithotrophic pregnancy (Stewart 2013). The dramatic changes required by these physiological adaptations for viviparous pregnancy involve significant changes to the transcriptome of the reproductive tract and the accompanying vasculature (Brandley et al. 2012; Girotti and Zingg 2003; V. L. Hansen et al. 2016; Hendrawan et al. 2017; Kidron et al. 1995; Ma et al. 2006; Nie, Li, Batten, et al. 2000; Smout et al. 2024). However, the molecular mechanisms of these cellular and physiological changes have been studied in only a few squamate taxa and are poorly known.

Studies of the uterine transcriptome in pregnancy have understandably placed considerable emphasis on mammalian models of pregnancy: therian mammals demonstrate sophisticated physiological adaptations to viviparous reproduction, and therian pregnancy has obvious significance to medical and veterinary interventions to improve reproductive health. Past studies have characterised changes in uterine gene expression accompanying pregnancy in mice and humans (Bethin et al. 2003), rats (Girotti and Zingg 2003), cows (Binelli et al. 2015), goats (Liu et al. 2020), pigs (Samborski et al. 2013), dogs (Zatta et al. 2017), and horses (Merkl et al. 2010) among others. However, while viviparity is ubiquitous in therian mammals, it is also widespread in squamate reptiles, with over 100 independent transitions to viviparity in this group (Whittington et al. 2022). The study of pregnancy in squamates is thus of particular interest to evolutionary biologists, as these animals provide an ideal model of a major evolutionary transition occurring many times independently, allowing for analysis of pregnancy in closely related viviparous and oviparous animals (Blackburn 2006).

The first transcriptomic study of a viviparous squamate, in the viviparous skink *Chalcides ocellatus*, suggested convergence in squamate and mammalian pregnancy at the level of differential gene expression (DGE) relating to tissue remodelling, immune regulation, angiogenesis and other pregnancy‐related processes in the pregnant uterus (Brandley et al. 2012). Later transcriptomic work on another viviparous skink, *Pseudemoia entrecasteauxii*, and the oviparous skinks *Lampropholis guichenoti* and *Lerista bougainvillii* indicated such gene expression changes are specific adaptations to viviparous pregnancy, with oviparous squamates showing more limited changes in uterine gene expression during gravidity (Griffith et al. 2016). A comparative study of uterine gene expression in the viviparous agamid lizard *Phrynocephalus vlangalii* and the closely related oviparous *P. przewalskii* (W. Gao et al. 2019) suggested a more complex picture, with significant downregulation of genes in the oviparous species during gravidity contrasting with greater upregulation of genes at the same stage of viviparous pregnancy, with oviparous and viviparous species showing distinct patterns of gene regulation. More recent comparative studies of oviparous and viviparous lineages of the reproductively bimodal lizard species *Zootoca vivipara* and *Saiphos equalis* (Foster et al. 2020; Recknagel et al. 2021) are of particular value in isolating differences in gene regulation related specifically to viviparous pregnancy and placing these transcriptomic changes in the context of known features of gene regulation in viviparous pregnancy in vertebrates. Collectively, these studies highlighted intriguing instances of convergence between squamates and therian mammals in uterine gene expression during viviparous pregnancy (Mika et al. 2022; Recknagel et al. 2021).

While the regulation of overall gene expression has long been recognised as vitally important to biological function, it is now understood that most genes produce multiple RNA transcripts due to a combination of alternatively spliced exons and alternative promoter usage, collectively termed differential transcript usage (DTU) (Boue et al. 2003; Pan et al. 2008; Yang et al. 2021). In many cases, this functions as an additional layer of gene regulation; alternative transcripts (or the resultant proteins) often show significant differences in stability and may be susceptible to nonsense‐mediated decay (NMD) (Soergel et al. 2013) or may lack key functional domains (Resch et al. 2004). This leads to important variance in gene function that may be hidden when considering only the total expression level of a given gene. The patterns of gene expression and alternative splicing tend to target different sets of functional genes, leading to both evolutionary and functional interactions between the two systems of gene regulation (Jacobs and Elmer 2020; Rogers et al. 2021). Alternative splicing is thus a key area of research for workers in the field of transcriptomics and gene regulation and in evolutionary biology (Singh and Ahi 2022; Verta and Jacobs 2022).

Alternative splicing has been shown to play a key role in processes related to viviparous pregnancy—for example, the vascular endothelial growth factor‐A (VEGF‐A) transcript, which functions as the main regulator of angiogenesis, has six major isoforms which differ in their biochemical properties and in the extent to which they stimulate endothelial cell proliferation. The importance of alternative splicing for viviparous pregnancy has been demonstrated many times in mammals, including humans (Ruano et al. 2021; Zeng et al. 2018), mice (Dabertrand et al. 2007; Yatsenko et al. 2004), rats (Gopalakrishnan and Kumar 2020), and rhesus monkeys (Kravitz et al. 2001). Although mammalian pregnancy has a distinct evolutionary origin from pregnancy in viviparous squamates, it seems highly likely that alternative splicing also plays a role in squamate pregnancy. However, to date, no published work has detailed the role of alternative splicing in pregnancy in any squamate species nor in a case of lecithotrophic viviparity.

In the present study, we characterise gene regulation across the female reproductive cycle during viviparous squamate pregnancy. Using long read sequencing to resolve expression and alternative transcript usage, we analyse a time series of immediately before pregnancy, during late‐stage pregnancy, and post‐parturition in the uterus of the common lizard, *Zootoca vivipara*. We identify and localise the regulation of genes that vary across stages and are thus shown to be involved in pregnancy and reproductive phases in this species. Our analyses include the differential expression and splicing of genes that are key candidates based on their roles in mammalian pregnancy or other squamate gene expression studies. We demonstrate fine‐scale molecular patterns of expression and splicing, finding a significant role for isoform switching in three candidate genes. Our results highlight the novel insights generated from both alternative splicing and gene expression together to understand the transcriptomic underpinnings of complex traits such as pregnancy. We provide new targets for further research on the functional genetics of pregnancy and viviparity in squamates.

## 2 Methods

### 2.1 Fieldwork and Animal Husbandry

Adult female *Z. vivipara* were caught from a large and stable wild population located at 55°47′33″–55°46′0″ N, 4°54′5″–4°56′4″ W, on the western side of Great Cumbrae, an island off the west coast of Scotland. Lizards were housed in naturalistic conditions in secure outdoor enclosures, exposed to natural temperatures and photoperiod, and provided with water and 1–3 beetle larvae ( *Tenebrio molitor* ) per lizard every 2–3 days for sustenance.

All lizards were held in common garden for at least 2 months before sampling. Pregnant females were identified by having a larger body girth and the presence of one or more mating‐bite marks on the ventral flanks near the lower leg. Lizards sampled during pregnancy were selected based on the ratio of weight to snout‐vent length (SVL) to ensure females at a similar stage of pregnancy were selected, which was later confirmed by embryo staging after dissection (Dufaure and Hubert 1961). Lizards for the post‐parturition stage were sampled 2–4 weeks post‐parturition. For the pre‐pregnancy stage, lizards were overwintered in captivity in a same‐sex enclosure and were then sampled shortly after emergence in spring.

### 2.2 Oviduct Sampling and RNA Extraction

We selected lizards for sampling at three different stages of the annual reproductive cycle: pre‐pregnancy (*n* = 2), late pregnancy (embryo stage 39–40 per Dufaure and Hubert 1961) (*n* = 4), and post‐parturition (*n* = 2). Late‐stage pregnancy was chosen as this is the stage at which the uterine transcriptome is likely to diverge most strongly: this is the point at which the demands of the developing embryo for water and calcium are at their highest and at which the deformation of the uterine tissues by large late‐stage embryos is the greatest (Lourdais et al. 2015; Stewart et al. 2009). Lizards were sacrificed in accordance with the Animals (Scientific Procedures) Act 1986 (Home Office 2013) Schedule 1 with the approval of the named veterinary surgeon (NVS) at the University of Glasgow (Ethics committee application number EA38/19) and of NatureScot (permit number 188744). From each female equivalent areas of the midsection of a single oviduct, approximately corresponding to the glandular uterus (where embryos are held during pregnancy) along with some material from the lower part of the infundibulum and upper part of the vagina, was dissected out and either used immediately for RNA extraction, or flash‐frozen in liquid nitrogen and stored at −70°C for later use.

RNA was extracted after tissue homogenisation in TRIzol (Zymo Research, Irvine US) and lysing using a FastPrep‐24 5G lysis system (MP Biomedicals, Irvine US) at speed 7 for two 30s intervals. We then extracted RNA using a standard commercial column RNA extraction kit (RNeasy Plus Mini Kit (Qiagen, Venlo NL) or Direct‐zol RNA Miniprep Kit (Zymo Research, Irvine US)) in accordance with the manufacturer's instructions. The quantity and purity (260/280 ratio) of the resulting RNA were checked using a Nanodrop spectrophotometer (Thermo Fisher Scientific, Waltham US) and integrity was checked by agarose gel electrophoresis. Purified total RNA samples were stored at −70°C until library preparation.

### 2.3 Library Preparation and Sequencing

We prepared barcoded cDNA libraries from uterine RNA samples with a PCR‐cDNA Barcoding Kit (ONT Ltd., Oxford, UK) according to the manufacturer's instructions. Libraries were checked for DNA quantity and purity using a Nanodrop spectrophotometer before adapter annealing and sequencing, confirming a nucleic acid concentration of at least 15 ng μL^−1^ and a 260/280 ratio of at least 1.7 for all samples. We loaded the prepared libraries onto R9.4.1 Flow Cells (ONT Ltd., Oxford UK) and sequenced using either a MinION portable sequencer or GridION benchtop sequencing device (both from ONT Ltd., Oxford UK).

We performed live basecalling using Guppy (Wick et al. 2019). After sequencing, we concatenated basecalled reads for each barcode, trimmed adapters using porechop (v0.2.4) applying an extra end trim of 20 bp (Wick et al. 2017), and filtered the trimmed reads with filtlong (v0.2.1) using a mean quality weight of 9 (default 1) and a target base threshold of 5,000,000,000,000 bp (*GitHub—Rrwick/Filtlong: Quality Filtering Tool for Long Reads*, n.d.). We then aligned reads to the *Z. vivipara* reference genome (RefSeq accession GCF_963506605.1) using minimap2 (v2.24) (Li 2018) and quantified transcripts from the aligned reads using salmon (v1.10.1) (Patro et al. 2017) running in ONT mode to accommodate long reads. When used in this way, Salmon does not use a quasi‐mapping approach, relying instead on alignments produced by minimap2 (Patro et al. 2017).

### 2.4 Data Analysis and Visualisation

We performed all statistical analyses using the statistical programming language R (v4.3.1) (R Core Team 2023). We first imported Salmon read quantification data with tximport (v1.30.0) (Soneson et al. 2015), and mapped transcript quantifications to gene counts using the *Z. vivipara* reference genome annotation (RefSeq accession GCF_963506605.1) with GenomicFeatures (v1.54.1) and AnnotationDbi (v1.64.1) (Lawrence et al. 2013). We then analysed DGE between pregnant and non‐pregnant samples, as well as between all conditions (pre‐pregnant, pregnant and post‐parturition) using DESeq2 (v1.48.2) (Love et al. 2014). In brief, we first filtered out genes with low levels of expression (quantified at less than 10 read counts in 2 or more samples), normalised the remaining genes by library size, estimated dispersions, fit a negative binomial GLM to calculate per‐gene log twofold changes (hereafter log FC) and Wald statistics, and adjusted for false discovery rate (FDR) using the Benjamini‐Hochberg method. Genes were considered differentially expressed where the calculated FDR‐adjusted *p*‐value (hereafter *p*‐adj) was lower than 0.1 and the absolute log FC was greater than 1. We also performed principal component analysis (PCA) on normalised gene counts.

Due to constraints on availability of biological material and our primary focus on pregnancy, our experiment used unequal sample sizes from pregnant (*n* = 4) as compared to pre‐pregnant and post‐parturition (*n* = 2) states. We therefore performed a subsampling analysis in which we equalised the sample size for the pregnant state, testing each possible permutation of *n* = 2 pregnant samples against either pre‐pregnancy or post‐parturition states, and repeated our differential expression analysis using the same approach described above. We then compared the results from subsamples to the results from main analysis (*n* = 4 pregnant samples) for the same comparisons and calculated Spearman's ρ for the resulting gene‐level data and compared the resulting sets of significant DEGs (see Supporting Information: Supplementary Results, Figures S4–S6).

Gene annotation was performed using the web‐based tool eggNOG‐mapper (http://eggnog‐mapper.embl.de) (Cantalapiedra et al. 2021; Huerta‐Cepas et al. 2019) to provide functional annotation and to clarify genes for which no gene symbol is provided in the *Z. vivipara* reference genome annotation (RefSeq accession GCF_011800845.1). eggNOG‐mapper assigns symbols, gene ontology (GO) terms and other annotations based on orthologs from pre‐computed gene trees for over 5000 genomes. Throughout the text, we have used the NCBI RefSeq gene symbols where available; where there is no gene symbol provided we have used the symbol as predicted by eggNOG‐mapper followed by the NCBI locus identifier in parentheses, for example H3F3A (LOC118083225). This is done to allow others to easily link the genes mentioned in the text to those in the current reference genome while still providing comprehensible information about gene identity for the reader.

We then carried out an over‐representation analysis (ORA) of biological process (BP) GO terms using topGO (2.54.0) (Alexa and Rahnenfuhrer 2023). In brief, we tested for over‐represented GO‐terms using Fishers Exact Test with the ‘weight’ algorithm, using gene lists of all significantly up‐ and downregulated genes for each comparison, against a gene universe of all expressed genes across all samples. We generated semantic space plots for all GO term sets using GO‐figure! (1.0.2) (Reijnders and Waterhouse 2021) based on our topGO results.

We also analysed differential transcript usage (DTU) using a two‐stage analysis with DEXSeq (v1.48.0) (Anders et al. 2012) and stageR (v1.24.0) (Van den Berge et al. 2017) following the methodology proposed by Love et al. (2018). We first used DRIMSeq (v1.30.0) (Robinson and Nowicka 2016) to filter out low‐expressed transcripts. We then used DEXSeq to normalise transcript counts for library size, estimate dispersions, and test for differential transcript usage for each gene. Finally, we used StageR to first screen for genes detected as participating in DTU by DEXSeq and then confirm which transcripts for each of these genes participate in DTU, using an overall false discovery rate (OFDR or alpha) of 0.05. We calculated Isoform Fraction (IF) values for all transcripts of all significant genes using IsoformSwitchAnalyseR (v2.2.0) (Vitting‐Seerup et al. 2019). We used Pfam (https://www.ebi.ac.uk/Tools/hmmer/search/hmmscan) (Mistry et al. 2021) to predict protein domains, SignalP (https://services.healthtech.dtu.dk/services/SignalP‐5.0/) (Teufel et al. 2022) to predict signal peptides, IUPred2A (https://iupred2a.elte.hu) (Dosztányi et al. 2005) to predict intrinsically disordered regions (IDRs) within transcripts, DeepTMHMM (https://dtu.biolib.com/DeepTMHMM/) (Hallgren et al. 2022) to predict protein topology, and DeepLoc2.0 (https://services.healthtech.dtu.dk/services/DeepLoc‐2.0/) (Thumuluri et al. 2022) to predict protein location within the cell for all transcripts of all significant genes.

Since the DTU analysis also used an unbalanced sample size for pregnancy (*n* = 4) as compared to pre‐pregnancy and post‐parturition (*n* = 2), we again performed a subsampling analysis in which we equalised the sample size for the pregnant state, testing each possible permutation of *n* = 2 pregnant samples in a similar manner to the DGE analysis. For each subsample, we performed DRIMSeq filtering, DEXSeq DTU analysis, and StageR. We then compared the results from subsamples to the results from the main analysis (*n* = 4 pregnant samples) for the same comparisons and calculated Spearman's ρ for the resulting transcript‐level data and compared the resulting sets of significant genes (see Supporting Information: Supplementary Results).

Figures were produced using ggplot2 (v3.4.4) (Wickham 2009) along with isoformSwitchAnalyseR for transcript plots, ggVennDiagram (v1.5.7) for Venn diagrams (C. H. Gao et al. 2024), and UpSetR (v1.4.0) for UpSet plots (Conway et al. 2017).

## 3 Results

### 3.1 Differential Gene Expression During Pregnancy

**FIGURE 1:**
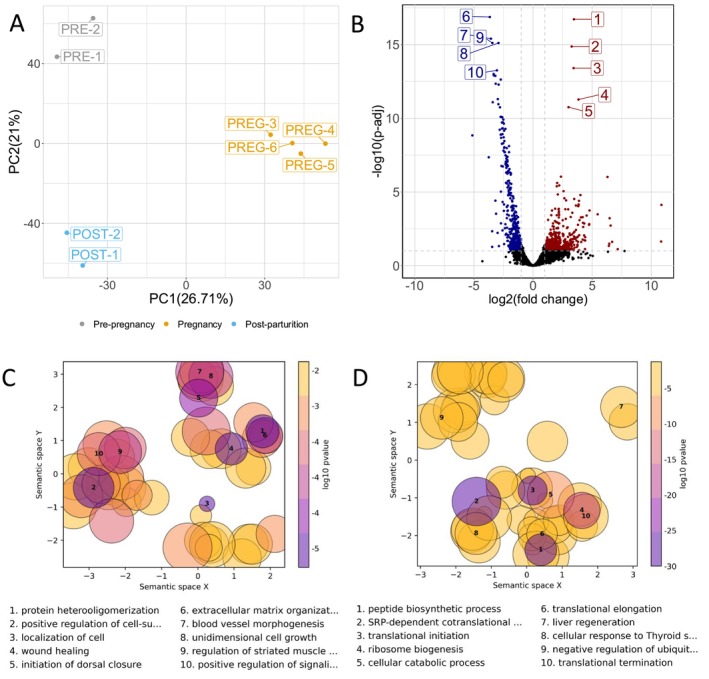
Differential gene expression during pregnancy in *Z. vivipara*. (A) Principal component analysis (PCA) of normalised gene expression in the uterus across three stages of the reproductive cycle in *Z. vivipara*. Two lizards were sampled pre‐pregnancy (PRE‐1 and PRE‐2, grey), four during pregnancy (PREG‐3–PREG‐6, gold) and two post‐parturition (POST‐1 and POST‐2, blue). The first two principal components are shown, together accounting for around 50% of the variation between samples. (B) Volcano plot showing differential gene expression in the uterus between pregnancy and pre‐pregnancy (upregulated genes are upregulated during pregnancy). Top upregulated genes are labelled (1–3) HBE1, (4) MALL, and (5) HBA. Top downregulated genes are labelled (6) RPS10, (7) ACER3, (8) RPLP1, (9) RPS18, and (10) RPS14. (C) Over‐represented Biological Processes Gene Ontology (BP:GO) terms for genes downregulated during pregnancy, plotted in semantic space. Top BP:GO terms are labelled with numbers and listed below. (D) Over‐represented BP:GO terms for genes downregulated during pregnancy as compared to post‐parturition, plotted in semantic space. Top BP:GO terms are labelled with numbers and listed below.

Median read length (N50) varied across sequencing runs from 913 bp–1.52 kbp and we obtained 626,822–2,222,284 reads per sample, with between 84.43%–94.17% of reads mapping successfully to the *Z. vivipara* reference genome (Table S1). Principal component analysis (PCA) showed well‐defined clustering of pre‐pregnancy, pregnant, and post‐parturition samples, with PC1 (26.74% of variation) clearly separating the pregnant and non‐pregnant samples whereas PC2 (21.00% of variation) separated pre‐pregnancy and post‐parturition samples, with pregnant samples occupying an intermediate position between pre‐pregnancy and post‐parturition samples (Figure 1A).

We first compared gene expression during pregnancy to all non‐pregnant states (pre‐pregnancy and post parturition combined) (Figure 1B). We found 874 differentially expressed genes (DEGs), of which 443 were upregulated and 431 were downregulated (Table S2). Top up‐ and downregulated DEGs included genes with a pre‐established connection to pregnancy, such as NOS1, MALL and CLCN2 (upregulated) and TUSC1 (downregulated) (Recknagel et al. 2021). Notably, the top 20 most strongly downregulated DEGs included 17 ribosomal genes: RPS10, RPLP1, RPS18, RPS14, RPS21, RPS12, RPS15, RPL13, RPL36, RPS27A, RPL12 (LOC LOC118084173), RPL31, RPS7, RPS8, RPL8, RPS23 (LOC118076902), and RPL6 (Table 1, Table S2). Of the 874 DEGs, 719 were also differentially expressed during pregnancy in at least one other viviparous amniote (based on data from Recknagel et al. 2021; Table S19).

**TABLE 1:** Top 20 differentially expressed genes in *Z. vivipara* uterus in pregnant stage vs. all non‐pregnant states. Listed is the gene symbol, log fold change, *p*‐value, false discovery rate (FDR)‐adjusted *p*‐values, and direction of regulation (up or down).

Gene	logFC	*p*	*p*‐adj	Direction
RPS10	−3.6604	3.38E‐21	1.33E‐17	Down
HBE1 (LOC118079427)	3.46156232	1.11E‐20	1.91E‐17	Up
ACER3	−3.5604703	2.98E‐19	3.92E‐16	Down
RPLP1	−2.9226536	7.44E‐19	7.71E‐16	Down
RPS18	−3.4364382	7.82E‐19	7.71E‐16	Down
HBE1 (LOC132592081)	3.26648159	1.52E‐18	1.33E‐15	Up
HBE1 (LOC118085601)	3.43122711	5.46E‐17	3.91E‐14	Up
RPS14	−3.0555792	9.14E‐17	5.55E‐14	Down
RPS21	−3.3326878	1.75E‐16	9.84E‐14	Down
RPS12	−3.317656	2.31E‐16	1.22E‐13	Down
RPS15	−3.1880338	2.66E‐16	1.31E‐13	Down
FAU	−2.7310586	5.06E‐16	2.35E‐13	Down
RPL13	−3.1588604	1.08E‐15	4.49E‐13	Down
RPL36	−2.9116696	1.30E‐15	5.13E‐13	Down
RPS27A	−2.9806144	1.33E‐14	4.99E‐12	Down
MALL	3.85018204	1.48E‐14	5.30E‐12	Up
STAT5B (LOC118094191)	−3.4085276	2.36E‐14	8.08E‐12	Down
RPL12 (LOC118084173)	−2.9289726	3.34E‐14	1.10E‐11	Down
HBA (LOC118092499)	3.00530993	5.81E‐14	1.76E‐11	Up
RPL31	−2.798116	6.36E‐14	1.79E‐11	Down

Over‐representation analysis of DEGs upregulated during pregnancy (Figure 1C) found several highly significant Biological Process Gene Ontology (BP:GO) terms connected to features with relevance to pregnancy, for example oxygen transport (GO:0015671), blood vessel morphogenesis (GO:0048514) and tissue remodelling processes (e.g., GO:0051674, GO:0042060, GO:0007392, GO:0030198) (Table S3). Top significant GO terms for downregulated DEGs (Figure 1D) were dominated by terms relating to ribosomal activity and protein translation (e.g., GO:0002181, GO:0006413, GO:0042254, GO:0006414, etc.) as well as protein transport and localisation (GO:0006614, GO:0006886) (Table S4).

### 3.2 Differential Gene Expression Across Reproductive Stages

**FIGURE 2:**
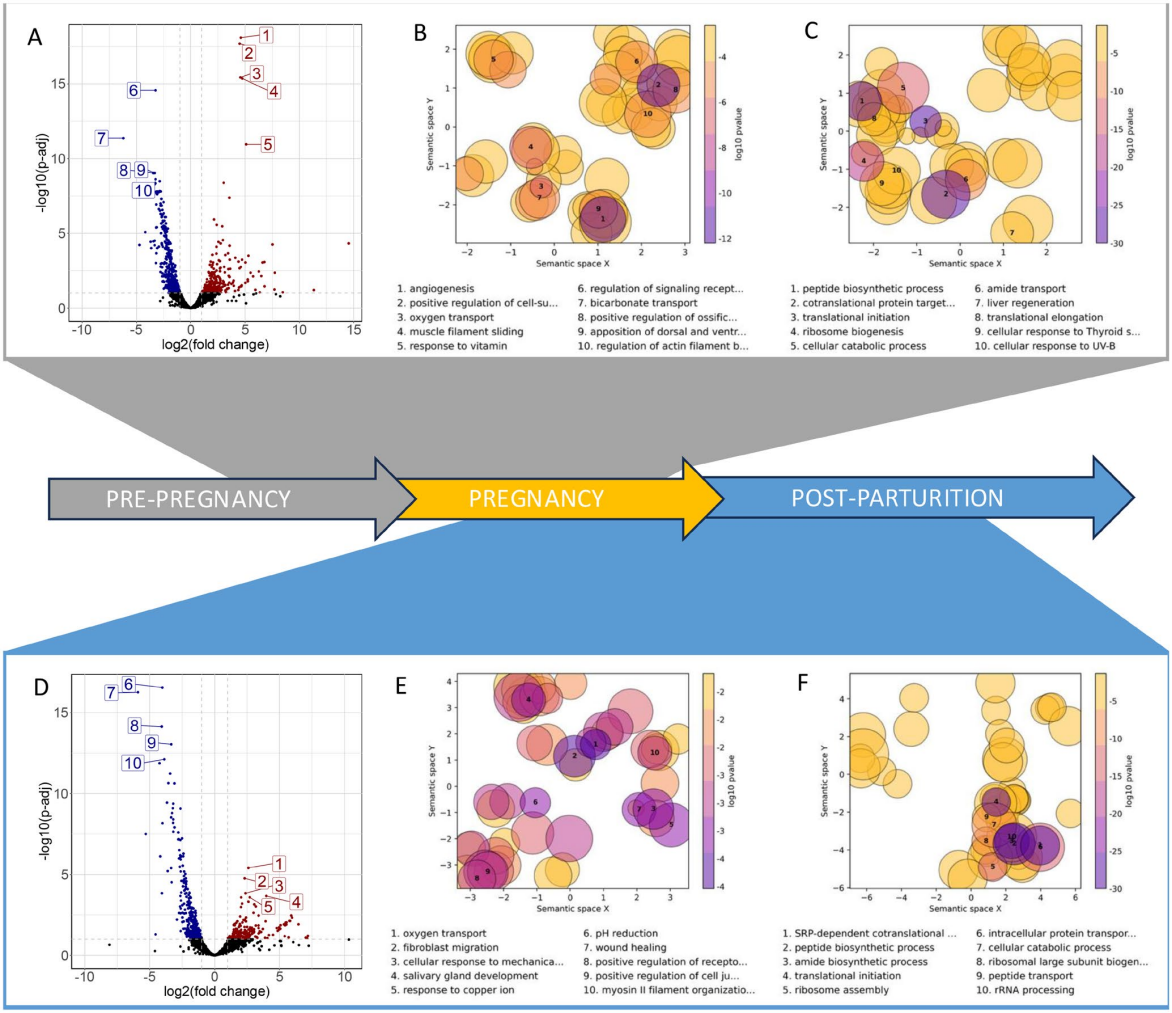
Differential gene expression before, during and after pregnancy in *Z*. *vivipara*. (A) Volcano plot showing differential gene expression in the uterus between pregnancy and pre‐pregnancy (upregulated genes are upregulated during pregnancy). Top upregulated genes are labelled (1–2) HBE1, (3) HBA, (4) HBE1, and (5) MALL. Top downregulated genes are labelled (6) ABCE1, (7) HSD17B11, (8) RPS12, (9) RPS18 and (10) RPS10. (B) Over‐represented Biological Process Gene Ontology (BP:GO) terms for genes upregulated during pregnancy as compared to pre‐pregnancy, plotted in semantic space. (C) Over‐represented BP:GO terms for genes downregulated during pregnancy as compared to pre‐pregnancy, plotted in semantic space. (D) Volcano plot showing differential gene expression in the uterus between pregnancy and post‐parturition (upregulated genes are upregulated during pregnancy). Top upregulated genes are labelled (1) CNN1, (2) HBE1, (3) DES, (4) CLDN7, and (5) CLU. Top downregulated genes are labelled (6) SURF1, (7) GAPDH, (8) RPS10, (9) RPLP1, and (10) ACER3. (E) Over‐represented BP:GO terms for genes upregulated during pregnancy as compared to post‐parturition, plotted in semantic space. (F) Over‐represented BP:GO terms for genes downregulated during pregnancy as compared to post‐parturition, plotted in semantic space.

We assessed changes in uterine gene expression along the time series of reproductive stages (Figure 2). 765 genes were differentially expressed between pre‐pregnancy and pregnancy stages (Table S5), including 299 upregulated during pregnancy and 466 downregulated (Figure 2A). Top differentially expressed genes are shown in Table 2. This total included 513 of the 874 genes identified as DEGs between pregnant and non‐pregnant uterus overall (i.e., when pooling all non‐pregnant samples, see previous section). Of the 765 DEGs, 707 were also differentially expressed during pregnancy in at least one other viviparous amniote (Table S19). Among the 299 upregulated genes, over‐representation analysis (Figure 2B,C) revealed BP:GO terms including angiogenesis (GO:0001525) and oxygen transport (GO:0015671), as well as terms relating to tissue remodelling processes (e.g., GO:0030198, GO:0010811, GO:0009611) and muscle‐cell related processes (e.g., GO:0030049 and GO:0007517) among the most significantly over‐represented GO terms (Table S6). For the 466 downregulated genes, the top over‐represented GO terms were again dominated by terms relating to ribosomal and protein translation processes (e.g., GO:0002181, GO:0043043, GO:0006413, GO:0042254, GO:0006414) (Table S7).

**TABLE 2:** Top 20 differentially expressed genes in *Z. vivipara* uterus in pregnant stage vs. pre‐pregnancy stage. Listed is the gene symbol, log fold change, *p*‐value, false discovery rate (FDR)‐adjusted *p*‐values, and direction of regulation (up or down).

Gene	logFC	*p*	*p*‐adj	Direction
HBE1 (LOC118079427)	4.61067155	4.34E‐22	8.09E‐19	Up
HBE1 (LOC132592081)	4.49110179	1.45E‐21	2.03E‐18	Up
HBA (LOC118092499)	4.55996353	3.26E‐19	3.65E‐16	Up
HBE1 (LOC118085601)	4.74625283	4.64E‐19	4.33E‐16	Up
ABCE1	−3.2427766	3.36E‐18	2.68E‐15	Down
HSD17B11 (LOC118084328)	−6.201856	9.24E‐15	4.31E‐12	Down
MALL	5.09760471	2.53E‐14	1.09E‐11	Up
RPS12	−3.4541882	2.99E‐12	9.28E‐10	Down
RPS18	−3.3108724	2.84E‐12	9.28E‐10	Down
RPS10	−3.2415161	8.03E‐12	2.37E‐09	Down
RPS17 (LOC118093187)	−2.856765	1.17E‐11	3.26E‐09	Down
LY6E (LOC118090479)	3.03510859	1.56E‐11	4.16E‐09	Up
ACER3	−3.2476626	1.78E‐11	4.51E‐09	Down
RPS21	−3.3121616	2.22E‐11	5.17E‐09	Down
RPL8	−2.8547361	6.74E‐11	1.51E‐08	Down
RPL35A	−2.886963	7.52E‐11	1.60E‐08	Down
RPL6	−2.7677295	8.29E‐11	1.60E‐08	Down
RPS14	−2.9222215	8.11E‐11	1.60E‐08	Down
RPS15	−3.0987047	7.82E‐11	1.60E‐08	Down
RPL13	−3.1184865	9.32E‐11	1.68E‐08	Down

When comparing pregnancy to post‐parturition, 554 genes were differentially expressed, including 189 genes upregulated during pregnancy and 362 downregulated (Figure 2D, Table S8). This included 407 genes that were also differentially expressed between pre‐pregnancy and pregnancy. The top 20 most differentially expressed genes were all downregulated during pregnancy (Table 3). Of the 554 DEGs, 508 were also differentially expressed during pregnancy in at least one other viviparous amniote (Table S19). Among the 189 upregulated DEGs, over‐represented BP:GO terms (Figure 2E) again included oxygen transport (GO:0015671) and terms relating to tissue remodelling processes (e.g., GO:0010761 and GO:0042060) and muscle cell related processes (GO:0031038, GO:1902905 and GO:0051153) (Table S9). For the 362 downregulated genes (Figure 2F), the most significant BP:GO terms again showed over representation of ribosomal and protein translation related processes (e.g., GO:0002181, GO:0006614 and GO:0043043) (Table S10).

**TABLE 3:** Top 20 differentially expressed genes in *Z. vivipara* uterus between pregnancy stage and post‐parturition stage. Listed is the gene symbol, log fold change, *p*‐value, discovery rate (FDR)‐adjusted *p*‐values, and direction of regulation (up or down).

Gene	logFC	*p*	*p*‐adj	Direction
SURF1 (LOC132591531)	−4.0317821	1.79E‐20	2.80E‐17	Down
GAPDH (LOC118087317)	−5.9029182	4.61E‐20	5.38E‐17	Down
RPS10	−4.0792839	7.64E‐18	7.15E‐15	Down
RPLP1	−3.3443509	1.15E‐16	8.95E‐14	Down
ACER3	−3.8732779	1.14E‐15	7.63E‐13	Down
RPL21 (LOC118076523)	−6.6659196	1.31E‐15	7.67E‐13	Down
STAT5B (LOC118094191)	−4.2444481	2.56E‐15	1.33E‐12	Down
RPL36	−3.4305797	1.36E‐14	5.78E‐12	Down
RPS18	−3.562004	5.87E‐14	2.29E‐11	Down
FAU	−3.089736	6.60E‐14	2.37E‐11	Down
RPL13A (LOC118085881)	−3.6662979	1.08E‐12	3.62E‐10	Down
RPS14	−3.1889368	1.35E‐12	4.20E‐10	Down
RPS7	−3.2183989	2.76E‐12	8.06E‐10	Down
LAMP1	−2.6299838	3.11E‐12	8.54E‐10	Down
RPS15	−3.2773628	6.13E‐12	1.59E‐09	Down
RPS21	−3.3532141	1.27E‐11	2.98E‐09	Down
RPS27A	−3.1852374	1.78E‐11	3.95E‐09	Down
RPL13	−3.1992343	3.17E‐11	6.44E‐09	Down
ST3GAL3	−4.0205801	3.46E‐11	6.74E‐09	Down
RPL12 (LOC118084173)	−3.0835514	6.89E‐11	1.24E‐08	Down

Finally, we compared the pre‐pregnancy uterus to post‐parturition uterus directly to characterise the starting and end phases of reproduction. We found 169 DEGs, of which 112 were upregulated and 57 were downregulated (Figure S1A, Table S11). Upregulated genes in the post‐parturition uterus relative to pre‐pregnancy showed over‐representation of BP:GO terms again including oxygen transport (GO:0015671) along with terms relating to immune system processes (e.g., GO:0048246 and GO:0042110) among others (Figure S1B, Table S12), whereas downregulated genes showed an overrepresentation of terms including skeletal muscle cell differentiation (GO:0035914) along with terms linked to virus replication processes (GO:190390 and GO:0044829) (Figure S1C, Table S13).

### 3.3 Expression of Candidate Genes for Amniote Pregnancy

We queried the expression of 38 candidate genes previously shown to be differentially expressed during viviparous pregnancy across multiple independent origins of amniote viviparity (Table S18) (Recknagel et al. 2021). Nineteen of these genes (i.e., 50%) were detected at significant levels in our sequencing experiment, of which two showed differential expression between pregnancy and non‐pregnancy (Figure S3): the Signal Sequence Receptor Subunit 3 SSR3 gene was downregulated (log FC −1.220, *p*‐adj = 0.0157), and the four and a half LIM domains 3 gene FHL3 was upregulated (log FC 2.186, *p*‐adj = 0.0575) during pregnancy. Both genes were also found to be differentially expressed during pregnancy in the Central Viviparous II lineage of *Z. vivipara* (Recknagel et al. 2021); we show they are similarly regulated in the widespread Western Viviparous lineage from which our samples originate.

### 3.4 Alternative Splicing Across Reproductive Stages

**FIGURE 3:**
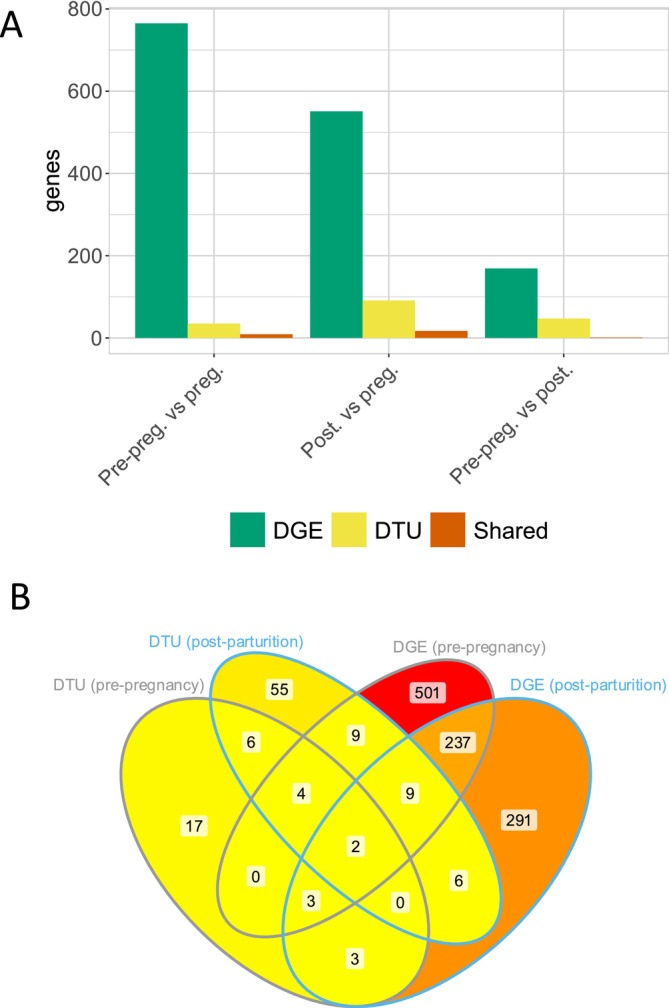
Comparison of differential gene expression (DGE) and differential transcript usage (DTU) during pregnancy in *Z. vivipara*. (A) Bar chart showing genes with evidence for DGE, DTU or both when comparing pregnancy to either pre‐pregnancy or post‐parturition states, and when comparing pre‐pregnancy and post‐parturition directly. (B) Venn diagram showing genes with evidence for DGE or DTU when comparing pregnancy to either pre‐pregnancy or post‐parturition states.

In addition to our analysis of differential gene expression, we looked for evidence of pregnancy‐related alternative splicing and alternative promoter use by analysing differential transcript usage (DTU) in the uterus between reproductive stages. Many fewer genes are involved in DTU than differential expression (DE) (Figure 3, Figure S2). We found 35 genes with evidence of DTU between pre‐pregnancy and pregnancy, of which nine were also significantly differentially expressed in the same comparison; 91 genes with DTU between pregnancy and post‐parturition, of which 17 were also differentially expressed; and 47 genes with DTU between pre‐pregnancy and post‐parturition, of which a single gene (TMBIM6) showed both differential expression and differential transcript usage (Figure 3).

Of the genes for which significant DTU was detected, 12 genes were shared between both the pre‐pregnancy/pregnancy and post‐parturition/pregnancy analyses: FAU, MARCHF3, MYL6, SRSF2, H3F3A (LOC118083225), PRPS2, NDUFB9, C8H6orf52, ATP5F1C, SRI, BLOC1S2, and LPP (Table S17). This list represents genes showing differences in isoform usage both between pre‐pregnancy and pregnancy and between pregnancy and post‐parturition, and so are of particular interest in terms of the transcriptomic regulation of pregnancy specifically and in terms of the potential for complex changes in isoform usage throughout the reproductive cycle. Of particular note are FAU and SRSF2, genes with an established role in mammalian pregnancy (Chwetzoff and D'Andrea 1997; Gu et al. 2015, 2018; Nie et al. 2002; Nie, Li, Batten, et al. 2000), and LPP, which was previously shown to be differentially expressed in pregnant viviparous *Z. vivipara* as compared to gravid oviparous *Z. vivipara* (Recknagel et al. 2021).

**FIGURE 4:**
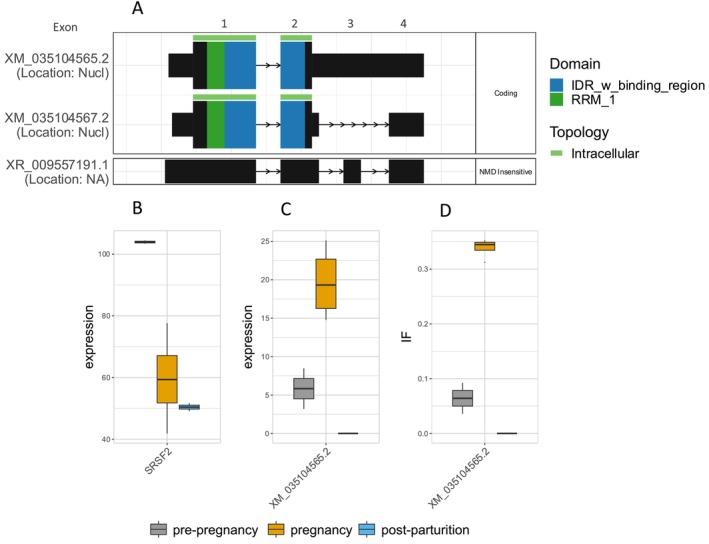
Isoform switching in SRSF2. (A) Diagram showing the three alternative transcripts of SRSF2 detected in this study. Exons are shown as solid blocks, with coding regions (CDS) shown as double thickness of untranslated regions (UTR). Colours denote protein domains: Blue for Intrinsically Disordered Regions (IDRs) with binding regions and green for RNA Recognition Motifs (RRM1) (B) Overall normalised expression levels of SRSF2 before, during and after pregnancy. (C) Normalised expression of transcript XM_035104565.2 of SRSF2 before, during and after pregnancy. (D) Isoform fraction (IF) of transcript XM_035104565.2 before, during and after pregnancy.

Three isoforms of SRSF2, a relevant candidate gene for pregnancy, were found at detectable levels: the protein‐coding mRNAs XM_035104565.2 and XM_035104567.2, which differ in the conformation of the 3′ untranslated region (UTR), and the alternative noncoding isoform XR_009557191.1. The two coding transcripts were both predicted to contain an RNA recognition motif (RRM) followed by an intrinsically disordered region (IDR) (Figure 4A). Expression of SRSF2 decreased slightly between pre‐pregnancy and pregnancy (log FC −0.807, *p*‐adj = 0.215), and again from pregnancy to post‐parturition (log FC −0.235, *p*‐adj 0.81960191) (Figure 4B), although the changes in expression were not statistically significant. Of the three isoforms, XM_035104565.2 showed significant participation in DTU during pregnancy when compared to both pre‐pregnancy and post‐parturition. Expression of the XM_035104565.2 transcript isoform actually increased during pregnancy despite the overall decrease in whole‐gene expression of SRSF2 (Figure 4C, Table S14). The isoform fraction (IF) XM_035104565.2 increased during pregnancy relative to pre‐pregnancy (∆IF 0.2958, *p*‐adj = 0.00034), before falling to undetectable levels post‐parturition (∆IF—0.184, *p*‐adj = 5.52E‐07) (Figure 4D, Table S15). Variation in the proportions of the other two isoforms of SRSF across reproductive stages was not statistically significant (*p*‐adj > 0.1).

**FIGURE 5:**
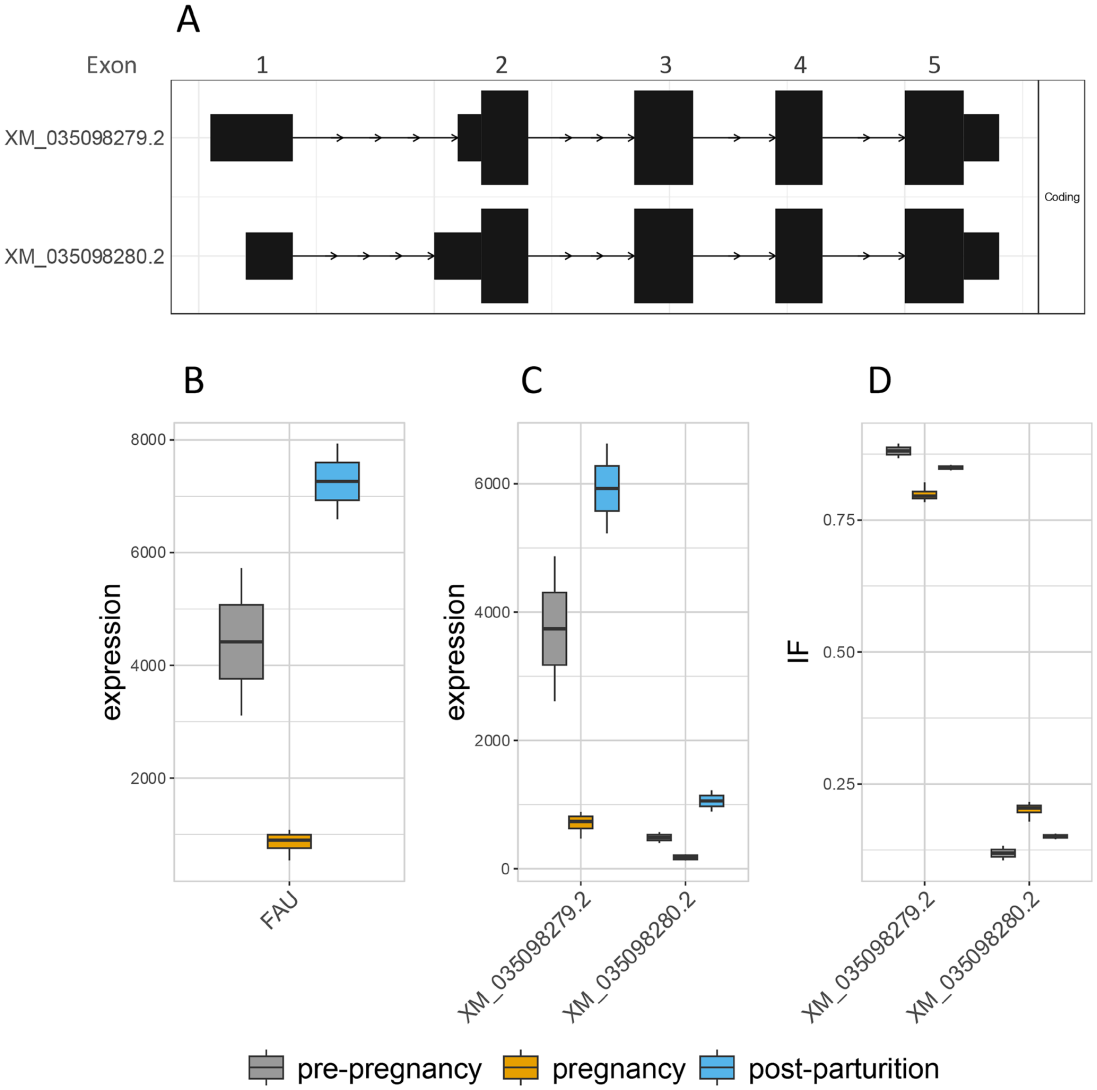
Isoform switching in FAU. (A) Diagram showing the two alternative transcripts of FAU detected in this study. Exons are shown as solid blocks, with coding regions (CDS) shown as double thickness of untranslated regions (UTR). (B) Overall normalised expression of FAU before, during and after pregnancy. (C) Normalised expression of each transcript of FAU before, during and after pregnancy. (D) Isoform fraction (IF) of each transcript of FAU before, during and after pregnancy.

Two isoforms of FAU were detected, XM_035098279.2 and XM_035098280.2. Both are annotated as proteincoding, although differing in the structure of the 5′ UTR (Figure 5A). Overall, FAU showed significant downregulation during pregnancy as compared to pre‐pregnancy (log FC −2.372, *p*‐adj = 8.19E‐07), followed by an increase in gene expression post‐parturition (log FC 3.090, *p*‐adj = 2.37E‐11) (Figure 5B), with both isoforms following this general pattern of expression (Figure 5C). However, subtle but significant shifts in the isoform fractions of both XM_035098279.2 and XM_035098280.2 were detected (Figure 5D), with the less abundant isoform XM_035098280.2 increasing in proportion between pre‐pregnancy and pregnancy (∆IF 0.082, *p*‐adj = 0.000693) (Table S14) before decreasing again post‐parturition (∆IF −0.050, *p*‐adj = 0.004021) (Table S15).

**FIGURE 6:**
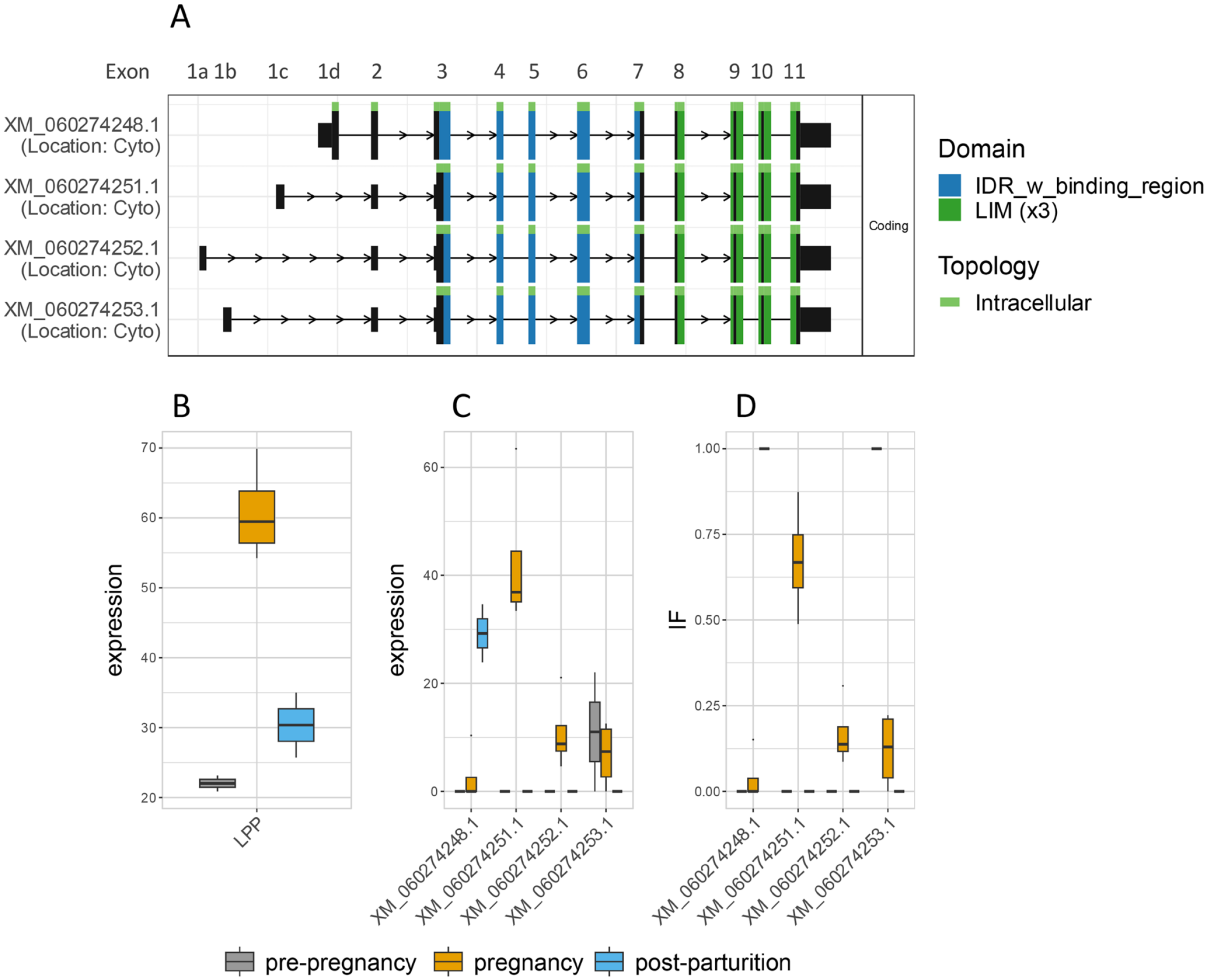
Isoform switching in LPP. (A) Diagram showing the four alternative transcripts of LPP detected in this study. Exons are shown as solid blocks, with coding regions (CDS) shown as double thickness of untranslated regions (UTR). Colours denote protein domains: Blue for Intrinsically Disordered Regions (IDRs) with binding regions and green for Lin‐11, Isl‐1 and Mec‐3 (LIM) domains. (B) Overall normalised gene expression of LPP before, during and after pregnancy. (C) Normalised expression of the four alternative transcripts of LPP before, during and after pregnancy. (D) Isoform fraction (IF) of the four alternative transcripts of LPP before, during and after pregnancy.

Four isoforms of LPP were detected, all annotated as protein coding mRNAs. All were predicted to code for intracellular proteins localised to the cytoplasm, containing an intrinsically disordered region (IDR) followed by three LIM domains (Figure 6A). The LPP gene was significantly upregulated during pregnancy when compared to pre‐pregnancy (log FC = 1.469, *p*‐adj = 0.0186) followed by a small, non‐significant decrease in expression post‐parturition (log FC = 0.994, *p*‐adj = 0.195) (Figure 6B). Expression of different isoforms, however, differed dramatically from the overall trend (Figure 6C,D). At pre‐pregnancy, the isoform XM_060274253.1 was the only transcript of LPP detected; during pregnancy, a mixture of all four isoforms was found in different proportions; and at post‐parturition, the isoform XM_060274248.1 was the only transcript detected (Tables S14 and S15).

## 4 Discussion

We characterised changes in gene expression and transcript usage in the uterus of pregnant *Z. vivipara* and found the involvement of genes that are both novel and shared across amniotes. Using a time series analysis at the three reproductive stages—before pregnancy, during pregnancy, and post‐parturition—for lizards held in a common environment, our analysis robustly focused on gestation whilst minimising as much as possible background effects such as season, growth, and population variation. We found a substantial effect of gestation and pregnancy on gene expression: 874 genes were differentially expressed during pregnancy compared to non‐pregnancy, with equivalent numbers of upregulated (443) and downregulated (431) genes. The most highly differentially expressed genes included several with a previously established connection to pregnancy across amniotes. We also separately analysed differential gene expression between pre‐pregnancy and pregnancy (765 genes) and between pregnancy and post‐parturition (554 genes) to characterise changes specific to each stage of the reproductive cycle.

Further, we show how changes to transcript isoform fractions resulting from alternative splicing varied with pregnancy and the reproductive cycle. To enable this analysis, we employed long‐read sequencing to characterise alternative isoforms with confidence (Verta and Jacobs 2022). Fewer genes were differentially spliced than had been differentially expressed overall; however, some differentially spliced genes were also differentially expressed, suggesting both pre‐ and post‐transcriptional regulation of the uterine transcriptome during pregnancy. We identified transcript isoforms that were specifically involved with pregnancy, including some that showed both differential expression and isoform switching during pregnancy. This interplay of differential expression and alternative isoform use demonstrates how these two layers of transcriptomic regulation respond flexibly across the phases of the reproductive cycle, as has been previously shown in mouse models (e.g., Dabertrand et al. 2007). Our study provides the first insights into the role of transcript isoform switching in viviparous pregnancy in a lecithotrophic squamate.

### 4.1 Shared Transcriptomic Basis of Pregnancy in *Z. vivipara* and Other Viviparous Amniotes

A key question for researchers studying the evolution of viviparity in vertebrates is the extent of molecular convergence: does the repeated evolution of the viviparous mode of reproduction also imply repeated changes to orthologous gene sequences, gene families, or molecular pathways? To date, the evidence for molecular convergence across independent transitions to viviparity remains equivocal. Evolutionary genomic analyses have generally shown weak or no evidence of molecular convergence in other contrasts (Eastment et al. 2024; Foster et al. 2022; Xie et al. 2025; Yusuf et al. 2023). Comparative transcriptomics suggests some convergence at the level of uterine gene expression, at least within amniotes (Brandley et al. 2012; Mika et al. 2022; Recknagel et al. 2021). Our results are consistent with this mixed picture, with evidence for convergence in the expression and/or alternative splicing of genes linked to viviparity in other amniotes, but also novel patterns of gene regulation without a strong preexisting link to pregnancy or viviparity.

Previous research on *Z. vivipara* and other lizard species has reported genes associated with pregnancy, and many of these were also significant in this study. Notably, one of the top significantly upregulated genes in pregnancy (compared to non‐pregnant states) was the Mal, T Cell Differentiation Protein Like gene (MALL), a member of the MAL proteolipid family. This gene has also been found to be upregulated during pregnancy in the viviparous African skink *Chalcides ocellatus* (Brandley et al. 2012) as well as in the viviparous Australian skink *Pseudemoia entrecasteauxii* (Griffith et al. 2016). NOS1, part of the nitrous oxide (NO) synthase family of genes, was significantly upregulated during pregnancy and has been previously shown to be differentially expressed in pregnant viviparous *Z. vivipara* as compared to gravid oviparous *Z. vivipara* (Recknagel et al. 2021). NO synthases have also been linked to pregnancy in mammals (H. Gao et al. 2009; Khorram et al. 1999; Welter et al. 2005; Yoshiki et al. 2000), possibly suggesting a shared role for NOS1 in the evolution of pregnancy in both mammals and squamates. Another upregulated gene in this analysis, the Chloride Voltage‐Gated Channel 2 gene (CLCN2), was also upregulated during pregnancy in two other viviparous squamates, *C. ocellatus* (Brandley et al. 2012) and the viviparous form of the reproductively bimodal Australian skink *Saiphos equalis* (Foster et al. 2020). The authors of the latter study also found this gene to be significantly differentially expressed during pregnancy versus gravidity when comparing viviparous and oviparous forms of *S. equalis* , suggesting a specific role for this gene in viviparous pregnancy in lecithotrophic squamates. Moreover, chloride channel proteins are also important for mammalian pregnancy: they are abundantly expressed in the human placenta, and expression seems to be increased in response to disease states such as pre‐eclampsia (Money et al. 2007; Murthi et al. 2012), possibly relating to their role in secretion (Duran et al. 2010). MALL, NOS1 and CLCN2 are therefore promising targets for future studies of gene function in pregnancy in viviparous squamates.

Intriguingly three of the most highly up‐regulated genes during pregnancy were orthologues of Haemoglobin Subunit Epsilon 1 gene (HBE1) (LOC118079427, LOC118085601 and LOC132592081). In mammalian models, HBE1 is normally expressed in the embryonic tissues very early in development, specifically in the earliest erythrocytes produced in the yolk sac. HBE1 is later supplanted first by the foetal haemoglobin genes expressed in the liver, and eventually by adult haemoglobins, in a process known as haemoglobin switching (Vinjamur et al. 2018). Little is known of the details of haemoglobin switching in squamate reptiles, although squamate haemoglobin genes are known to differ substantially from those of mammals in terms of both copy number and expression (Hoffmann et al. 2018). The possible expression of ‘embryonic’ type haemoglobin genes in the adult uterus during lizard pregnancy is unexpected and deserves further study, given the importance of oxygen transport in late‐stage pregnancy when the oxygen demands of the embryo are at their highest (Sartori et al. 2017).

Suppression of cancer associated genes is important in mammalian pregnancy to prevent miscarriages, and we see parallels to this in viviparous squamates. We found that the Tumour Suppressor Candidate 1 gene (TUSC1) was strongly downregulated in pregnancy; TUSC1 is a tumour suppressor gene, but high TUSC1 expression in the endometrium has been linked to miscarriages in humans (Ran et al. 2022). Further study of the role of TUSC1 downregulation in successful pregnancy is certainly warranted. This result parallels the downregulation of the Tumour Necrosis Factor gene (TNF) seen during pregnancy in the skink *Pseudemoia entrecasteauxii* (Hendrawan et al. 2017). TNF is another cancer‐related gene with a link to miscarriages in humans (Sudhir et al. 2016). TNF itself was not significantly downregulated during pregnancy in our study, instead showing very low or undetectable levels of expression at all reproductive stages, although the functionally related TNF Alpha Induced Protein 2 gene (TNFAIP2) was downregulated in the pregnant uterus.

A particularly striking result was the very high number of ribosomal genes which were found to be downregulated during pregnancy. The top 20 most downregulated genes for pregnancy relative to non‐pregnancy included 17 ribosomal protein genes (Table S2), and the seven most significant Biological Process GO terms for genes downregulated during pregnancy all referred to protein translation or ribosome synthesis (Table S4). Ribosomal proteins are generally highly expressed across many cell types, as they are essential for ribosome synthesis, and thus by extension all cellular life. This unexpected downregulation of ribosomal protein genes is not unique to *Z. vivipara*; in the skink *C. ocelatus*, ribosomal protein genes were also significantly downregulated during pregnancy (Brandley et al. 2012). Nor is the phenomenon unique to squamates; ribosomal protein genes also appear to be suppressed in the uterus of the rat *Rattus norvegicus* during pregnancy (Girotti and Zingg 2003). Other reports, however, point to upregulation of ribosomal genes in mammalian pregnancy, especially around early pregnancy and implantation (Kidron et al. 1995; Ma et al. 2006). We speculate that the downregulation of ribosomal genes in pregnancy that we identified is due to upregulation of ribosomal processes in the pre‐pregnancy and post‐parturition stages, rather than suppression in the pregnant uterus per se. Such regulatory dynamics may be required in preparation for the transcriptomic changes during pregnancy in the uterus, and recovery from pregnancy during the post‐parturition phase, given the mechanical and other stresses involved in viviparous pregnancy. Alternatively, the suppression of ribosomal protein gene expression during late pregnancy may be a direct product of the stresses placed on the tissues of the uterus at this point in pregnancy, when the volume of the embryo and its demands for water and oxygen are at their most extreme (Bonnet et al. 2017; Sartori et al. 2017). Further research is required to explore this pattern of ribosomal protein gene downregulation and to investigate its role in viviparous pregnancy in *Z. vivipara* and other species.

### 4.2 Alternative Splicing and Alternative Promoter Use in Pregnancy in *Z. vivipara*

We show a significant role for alternative splicing both in the transition from pre‐pregnancy to pregnancy and from pregnancy to post‐parturition in *Z. vivipara*. Given the significance of alternative splicing in mammalian pregnancy (Gopalakrishnan and Kumar 2020; Nie, Li, Batten, et al. 2000; Ruano et al. 2021) and the absence of published data on the same process in viviparous pregnancy in squamates, these results help to fill in an important gap in our understanding of the transcriptomics of viviparous pregnancy across amniotes.

Our analysis of differential transcript usage in *Z. vivipara* before, during and after pregnancy produced contrasting results to our analysis of gene expression. Whereas the largest difference in gene expression was observed between pre‐pregnancy and pregnancy stages, this comparison saw the fewest genes with DTU; the highest rates of DTU were seen between the pregnancy and post‐parturition stages (Figure 3). Furthermore, many genes undergoing DTU were not significant in our differential gene expression (DGE) analysis (Figure 3). This result highlights the importance of considering both DGE and DTU when investigating the transcriptomic basis of complex traits, where regulation of many genes is implicated in determining phenotype (Jacobs and Elmer 2020). Our findings also demonstrate the potential of long‐read RNA sequencing in comprehensively describing a full range of gene regulation through both differential expression and alternative splicing.

Although previous studies of squamate pregnancy have not addressed alternative splicing directly, we note that one gene that we found to exhibit a complex pattern of both differential expression and alternative splicing during pregnancy was previously reported as differentially expressed between viviparous and oviparous *Z. vivipara* (Recknagel et al. 2021): the LIM Domain Containing Preferred Translocation Partner In Lipoma gene (LPP). LPP is a gene with a complex cellular role. First characterised in connection with lipomas (Petit et al. 1996), LPP has been suggested as a mediator of cell–cell adhesion through interaction with cell junctions and the cytoskeleton (Hansen and Beckerle 2006, 2008), as a scaffold protein for the assembly of other protein complexes, and as a potential regulator of gene expression (Petit et al. 2000). Alternative isoforms of LPP have been reported to localise in the testes of male mice (Vervenne et al. 2009) and in diseased cells of gastric cancer and hypoxic–ischaemic encephalopathy (Jin et al. 2022; Xue et al. 2020). Isoform switching in this gene has not previously been reported for uterine tissues in connection with pregnancy.

Our findings strongly suggest that alternative promoter use is the splicing mechanism underlying DTU in LPP. All four isoforms of LPP we detected (three short, one long) were cytoplasmic and differed primarily in using alternative first exons. The short variants show a truncated version of exon 3, in which the coding sequence starts part of the way through the exon (Figure 6A). These short forms of LPP appear to predominate before and during pregnancy, whereas only the longer form was expressed post‐parturition (Figure 6C,D). All transcript variants also had different 5′ UTR sequences, which, given the role of the 5′ UTR in downstream regulation of mRNA translation (Araujo et al. 2012; Ryczek et al. 2023; Van Der Velden and Thomas 1999), may have significant implications for LPP protein expression levels at different stages of the reproductive cycle. Therefore we speculate that this truncation of exon 3 has functional significance linked to pregnancy in *Z. vivipara*. To our knowledge this is the first time that alternative splicing of LPP has been implicated in pregnancy for any animal, and further investigation of the role of alternate isoforms of this gene in pregnancy in *Z. vivipara* and other amniotes is needed.

### 4.3 Alternative Splicing of Implantation Genes in *Z. vivipara* Pregnancy

Among the candidates showing differential transcript usage before, during and after pregnancy in *Z. vivipara*, two genes had been previously linked to pregnancy in mammals: the Serine And Arginine Rich Splicing Factor 2 gene (SRSF2) and the FAU Ubiquitin Like And Ribosomal Protein S30 Fusion gene (FAU).

SRSF2 is a key component of the spliceosome (Kramer 1996) and an important regulator of alternative splicing in a range of biological contexts (H.‐H. Chen et al. 2006; Komeno et al. 2013; Lei et al. 2023; Wang et al. 2001). SRSF2 is ubiquitously expressed in most tissues (Fagerberg et al. 2014) and plays a critical role in early pregnancy in both primates and mice, specifically in implantation (Nie et al. 2002; Nie, Li, Batten, et al. 2000; Salamonsen et al. 2002). Implantation is a complex, multi‐stage process involving the attachment of the mammalian blastocyst to the endometrium, and, in rodents, primates, and certain other placental mammals, the invasion of the embryo into the uterine wall (Chavatte‐Palmer and Guillomot 2007; McGowen et al. 2014). SRSF2 is strongly upregulated at implantation sites during early pregnancy in mice (Nie, Li, Batten, et al. 2000), as well as in primates during the secretory phase of the oestrous cycle (Nie et al. 2002), when the mammalian uterus becomes receptive to embryo implantation.

Alternative splicing of SRSF2 is implicated in mammalian pregnancy: four transcript isoforms have been identified in the murine uterus with distinct conformations of the 3′ UTR (Nie, Li, Batten, et al. 2000). Changes to the 3′ UTR of SRSF2 transcripts have been shown to alter the half‐life, translational efficiency, and localisation of mRNAs (Sureau and Perbal 1994), with potential ramifications for splicing of a host of other transcripts acted on by the spliceosome. The isoform XM_035104565.2, which we found showed significant variance in usage, differed from the alternative coding mRNA XM_035104567.2 specifically in the 3′ UTR (Figure 4A). This suggests a role for alternative splicing of the 3′ UTR sequence of this gene in the overall regulation of transcript splicing in pregnancy in *Z. vivipara*, suggesting deep homology or evolutionary convergence in the function of SRSF2 in viviparous squamate and mammalian pregnancy (Recknagel et al. 2021; Van Dyke et al. 2014).

FAU is another gene we identified in our DTU analysis which has been linked to mammalian pregnancy, and specifically implantation (Chwetzoff and D'Andrea 1997; Gu et al. 2015, 2018; Nie, Li, Hampton, et al. 2000). The protein encoded by FAU is a fusion protein comprising the ubiquitin‐like protein FUBI and the ribosomal protein S30, with the resulting protein product cleaved to yield both proteins (Kas et al. 1992; van den Heuvel et al. 2021). The FUBI protein is a subunit of the MNSF protein complex, which functions as a nonspecific suppressor of B‐ and T‐cell‐mediated immune response (Nakamura et al. 1987), and its role in implantation and pregnancy in mammals is likely linked to modulation of the maternal immune response in the endometrium to prevent rejection of the embryo (Nie, Li, Hampton, et al. 2000). In contrast to SRSF2, FAU has been shown to be downregulated at implantation sites in the murine uterus (Nie, Li, Hampton, et al. 2000).

FAU appears strongly downregulated in the pregnant *Z. vivipara* uterus (Figure 5B). Additionally, this downregulation is accompanied by a subtle but significant shift in the isoform fraction of the two alternatively spliced transcripts of FAU, with the less abundant transcript XM_035098280.2, containing a different conformation of the 5′ UTR, showing a slight but consistent increase in isoform fraction. Since such modifications to the 5′ UTR can play a major role in downstream regulation of transcripts (Araujo et al. 2012; F. Chen et al. 2022; Lin and Li 2012; Wieder et al. 2024), this shift in isoform usage may accompany subtle changes in expression of the FUBI and ribosomal S30 proteins, potentially contributing to the FAU downregulation seen here (Figure 5B). It is notable that mouse model studies of the role of FAU in the endometrium during implantation also recovered isoforms of FAU that differed in the 5′ UTR (Nie, Li, Hampton, et al. 2000), suggesting that changes to this region may also be functionally relevant to pregnancy in mammals. In the case of *Z. vivipara*, it is unclear whether this pattern of expression and isoform usage is driven primarily by regulation of the FUBI MNSF complex protein, or by the general pattern of downregulation of multiple ribosomal genes acting on the S30 ribosomal protein (e.g., Table 1, Supplementary Table S2), or a combination of the two processes.

Invasive implantation, in which the embryo forms specialised placental structures within the uterine wall, is a derived process which is generally thought to be specific to mammalian pregnancy (Blackburn 2006). Although invasive implantation has been proposed for at least one lizard species (Blackburn and Flemming 2011), squamates generally exhibit a far less complex placental morphology with relatively simple apposition of the embryonic and uterine epithelium, as in the case of lecithotrophic viviparity in *Z. vivipara* (Stewart et al. 2004). The patterns of whole gene expression for the implantation genes SRSF2 and FAU observed in this study do not precisely match those seen at implantation sites in mouse models of pregnancy: whereas FAU was downregulated in both, SRSF2 was downregulated in *Z. vivipara* but upregulated in mice. The expression patterns described in mice were observed at the start of pregnancy during initial attachment of the embryo to the implantation site (Nie, Li, Hampton, et al. 2000), whereas our study addresses late‐stage pregnancy in *Z. vivipara*. However, we note that researchers studying marsupial implantation and parturition have argued that implantation in eutherian mammals may involve homologous patterns of gene expression to those seen immediately prior to parturition in marsupials, with the latter likely representing the ancestral form of mammalian pregnancy (Griffith et al. 2017). Given that both SRSF2 and FAU have been shown to play a role in inflammation (Gerlevik et al. 2024; Nakamura and Watanabe 2010; Pollyea et al. 2019), we speculate that DTU involvement for these genes in late‐stage pregnancy in *Z. vivipara* may be connected to the regulation of inflammatory processes as part of the modulation of the innate immune system to facilitate extended gestation.

Given these differences in expression patterns, we expect that these genes likely play a somewhat different role in pregnancy in *Z. vivipara* as compared to mice or other mammals. However, given their significance for pregnancy across amniotes, the functional role of expression and alternative splicing of these genes during pregnancy is an important candidate for future research.

## 5 Conclusions

We present a study of gene expression throughout reproductive stages in the widespread viviparous lizard *Z. vivipara*, an important emerging model organism for research on the evolution of viviparous pregnancy. Further, we provide novel evidence of the role of alternative splicing and alternative promoter use in lecithotrophic viviparous pregnancy. We have identified evidence of convergence at the level of gene regulation between *Z. vivipara* and other viviparous amniotes, as well as changes in gene regulation not previously linked explicitly to viviparity. These results further reinforce the importance of alternative splicing in viviparous pregnancy, a molecular mechanism already well evidenced to play a role in mammals but previously unknown in squamates.

Future transcriptomic studies of viviparous pregnancy in squamates will show whether the patterns of alternative splicing and alternative promoter use seen in *Z. vivipara* are mirrored in other viviparous lineages, or whether this layer of gene regulation is more lineage‐specific. While our results indicate a role for alternative splicing and alternative promoter use in squamate pregnancy, how these transcriptomic changes relate to the physiology of pregnancy remains an open question: functional genetic experiments are needed to explore how different isoforms of these genes influence protein structure, half‐life, and function to explain the link between isoform switching and pregnancy in cases of lecithotrophic viviparity. Our findings lay the groundwork for future experiments to characterise the function of pregnancy‐related genes in *Z. vivipara* and other viviparous amniotes.

## Author Contributions

K.R.E., M.M.B., M.M. and J.L.S. designed the experiment. K.R.E. and J.L.S. conducted field work. J.L.S. conducted the molecular laboratory work, sequencing, data analysis, and visualisation. J.L.S. wrote the manuscript with input from K.R.E. All authors edited and approved the manuscript.

## Funding

This research was supported by a University of Glasgow College of Medical, Veterinary and Life Sciences‐Doctoral Training Programme studentship to J.L.S. with K.R.E., M.M.B. and M.M. This study was also supported by NERC grants NBAF964, NBAF1018, NE/N003942/1 and NE/V001728/1.

## Conflicts of Interest

The authors declare no conflicts of interest.

## Supporting information

Supplementary Results: Differential gene expression sensitivity analysis, Differential transcript usage sensitivity analysis.
**Figure S1:** Differential gene expression before and after pregnancy in *Z. vivipara*.
**Figure S2:** Comparison of differential gene expression (DGE) and differential transcript usage (DTU) during pregnancy in *Z. vivipara*.
**Figure S3:** Boxplots showing expression levels (normalised read counts) of two candidate genes differentially expressed during pregnancy in the *Z. vivipara* uterus.
**Figure S4:** Significant DEGs when subsampling pregnant uterus samples to equalise sample size.
**Figure S5:** Genes with evidence of significant differential transcript usage when subsampling pregnant uterus samples to equalise sample size.
**Figure S6:** Principal component analysis (PCA) of transcript‐level read counts after filtering and normalisation.

**Table S1:** Sample sheet.
**Table S2:** DEGs preg‐non.
**Table S3:** GOs preg‐non up.
**Table S4:** GOs preg‐non down.
**Table S5:** DEGs preg‐pre.
**Table S6:** GO terms preg‐pre up.
**Table S7:** GOs preg‐pre down.
**Table S8:** DEGs preg‐post.
**Table S9:** GOs preg‐post up.
**Table S10:** GOs preg‐post down.
**Table S11:** DEGs post‐pre.
**Table S12:** GOs post‐pre up.
**Table S13:** GOs post‐pre down.
**Table S14:** DTU pre‐preg.
**Table S15:** DTU preg‐post.
**Table S16:** DTU post‐pre.
**Table S17:** DTU shared.
**Table S18:** Candidate genes.
**Table S19:** Amniote genes.
**Table S20:** DGE subsampling.
**Table S21:** DTU subsampling.

## Data Availability

Sequencing results have been archived with NCBI (BioProject PRJNA1256845). Additionally, we have archived all relevant scripts, intermediate files and other Supporting Information for our study on University of Glasgow Enlighten repository with the following permanent DOI: https://doi.org/10.5525/gla.researchdata.1950.

## References

[mec70469-bib-0001] Alexa, A. , and J. Rahnenfuhrer . 2023. “topGO: Enrichment Analysis for Gene Ontology.” 10.18129/B9.bioc.topGO.

[mec70469-bib-0002] Anders, S. , A. Reyes , and W. Huber . 2012. “Detecting Differential Usage of Exons From RNA‐Seq Data.” Genome Research 22, no. 10: 2008. 10.1101/GR.133744.111. 22722343 PMC3460195

[mec70469-bib-0003] Andrews, R. M. 1997. “Evolution of Viviparity: Variation Between Two Sceloporine Lizards in the Ability to Extend Egg Retention.” Journal of Zoology 243, no. 3: 579–595. 10.1111/J.1469-7998.1997.TB02803.X.

[mec70469-bib-0004] Araujo, P. R. , K. Yoon , D. Ko , et al. 2012. “Before It Gets Started: Regulating Translation at the 5′ UTR.” International Journal of Genomics 2012, no. 1: 475731. 10.1155/2012/475731. PMC3368165 22693426

[mec70469-bib-0005] Bethin, K. E. , Y. Nagai , R. Sladek , et al. 2003. “Microarray Analysis of Uterine Gene Expression in Mouse and Human Pregnancy.” Molecular Endocrinology 17, no. 8: 1454–1469. 10.1210/ME.2003-0007. 12775764

[mec70469-bib-0006] Binelli, M. , S. C. Scolari , G. Pugliesi , et al. 2015. “The Transcriptome Signature of the Receptive Bovine Uterus Determined at Early Gestation.” PLoS One 10, no. 4. 10.1371/JOURNAL.PONE.0122874. PMC4388694 25849079

[mec70469-bib-0007] Blackburn, D. G. 1992. “Convergent Evolution of Viviparity, Matrotrophy, and Specializations for Fetal Nutrition in Reptiles and Other Vertebrates.” Integrative and Comparative Biology 32, no. 2: 313–321. 10.1093/ICB/32.2.313.

[mec70469-bib-0008] Blackburn, D. G. 2006. “Squamate Reptiles as Model Organisms for the Evolution of Viviparity.” Herpetological Monographs 20, no. 1: 131–146. 10.1655/0733-1347(2007)20.

[mec70469-bib-0009] Blackburn, D. G. , and A. F. Flemming . 2010. “Reproductive Specializations in a Viviparous African Skink: Reproductive Specializations in a Viviparous African Skink: Implications for Evolution and Biological Conservation Implications for Evolution and Biological Conservation.” https://digitalrepository.trincoll.edu/facpub.

[mec70469-bib-0010] Blackburn, D. G. , and A. F. Flemming . 2011. “Invasive Implantation and Intimate Placental Associations in a Placentotrophic African Lizard, *Trachylepis ivensi* (Scincidae).” Journal of Morphology 273, no. 2: 137–159. 10.1002/JMOR.11011. 21956253

[mec70469-bib-0011] Bonnet, X. , G. Naulleau , and R. Shine . 2017. “The Evolutionary Economics of Embryonic‐Sac Fluids in Squamate Reptiles.” American Naturalist 189, no. 3: 333–344. 10.1086/690119/ASSET/IMAGES/LARGE/FG3.JPEG. 28221829

[mec70469-bib-0012] Boue, S. , I. Letunic , and P. Bork . 2003. “Alternative Splicing and Evolution.” BioEssays 25, no. 11: 1031–1034. 10.1002/BIES.10371. 14579243

[mec70469-bib-0013] Brandley, M. C. , R. L. Young , D. L. Warren , M. B. Thompson , and G. P. Wagner . 2012. “Uterine Gene Expression in the Live‐Bearing Lizard, Chalcides Ocellatus, Reveals Convergence of Squamate Reptile and Mammalian Pregnancy Mechanisms.” Genome Biology and Evolution 4, no. 3: 394–411. 10.1093/gbe/evs013. 22333490 PMC3318437

[mec70469-bib-0014] Cantalapiedra, C. P. , A. Hern̗andez‐Plaza , I. Letunic , P. Bork , and J. Huerta‐Cepas . 2021. “eggNOG‐Mapper v2: Functional Annotation, Orthology Assignments, and Domain Prediction at the Metagenomic Scale.” Molecular Biology and Evolution 38, no. 12: 5825–5829. 10.1093/molbev/msab293. 34597405 PMC8662613

[mec70469-bib-0015] Chavatte‐Palmer, P. , and M. Guillomot . 2007. “Comparative Implantation and Placentation.” Gynecologic and Obstetric Investigation 64, no. 3: 166–174. 10.1159/000101742. 17934314

[mec70469-bib-0016] Chen, F. , M. Cocaign‐Bousquet , L. Girbal , and S. Nouaille . 2022. “5′ UTR Sequences Influence Protein Levels in *Escherichia coli* by Regulating Translation Initiation and mRNA Stability.” Frontiers in Microbiology 13: 1088941. 10.3389/FMICB.2022.1088941/BIBTEX. 36620028 PMC9810816

[mec70469-bib-0017] Chen, H.‐H. , Y.‐C. Wang , and M.‐J. Fann . 2006. “Identification and Characterization of the CDK12/Cyclin L1 Complex Involved in Alternative Splicing Regulation †.” Molecular and Cellular Biology 26, no. 7: 2736–2745. 10.1128/MCB.26.7.2736-2745.2006. 16537916 PMC1430317

[mec70469-bib-0018] Chwetzoff, S. , and S. D'Andrea . 1997. “Ubiquitin Is Physiologically Induced by Interferons in Luminal Epithelium of Porcine Uterine Endometrium in Early Pregnancy: Global RT‐PCR cDNA in Place of RNA for Differential Display Screening 1.” FEBS Letters 405, no. 2: 148–152. 10.1016/S0014-5793(97)00177-4. 9089280

[mec70469-bib-0019] Conway, J. R. , A. Lex , and N. Gehlenborg . 2017. “UpSetR: An R Package for the Visualization of Intersecting Sets and Their Properties.” Bioinformatics 33, no. 18: 2938–2940. 10.1093/BIOINFORMATICS/BTX364. 28645171 PMC5870712

[mec70469-bib-0020] Dabertrand, F. , N. Fritz , J. Mironneau , N. Macrez , and J. L. Morel . 2007. “Role of RYR3 Splice Variants in Calcium Signaling in Mouse Nonpregnant and Pregnant Myometrium.” American Journal of Physiology—Cell Physiology 293, no. 3: 848–854. 10.1152/AJPCELL.00069.2007/ASSET/IMAGES/LARGE/ZH00090753510006.JPEG. 17596299

[mec70469-bib-0021] Dosztányi, Z. , V. Csizmok , P. Tompa , and I. Simon . 2005. “IUPred: Web Server for the Prediction of Intrinsically Unstructured Regions of Proteins Based on Estimated Energy Content.” Bioinformatics 21, no. 16: 3433–3434. 10.1093/BIOINFORMATICS/BTI541. 15955779

[mec70469-bib-0133] Dufaure, J. P. , and J. Hubert . 1961. “Table de développement du lézard vivipare: Lacerta (Zootoca) vivipara.” Archives d’Anatomie Microscopique et de Morphologie Experimentale 50: 309–328.

[mec70469-bib-0022] Duran, C. , C. H. Thompson , Q. Xiao , and H. C. Hartzell . 2010. “Chloride Channels: Often Enigmatic, Rarely Predictable.” Annual Review of Physiology 72: 95–121. 10.1146/ANNUREV-PHYSIOL-021909-135811. PMC2851227 19827947

[mec70469-bib-0023] Eastment, R. V. , B. B. M. Wong , and M. D. McGee . 2024. “Convergent Genomic Signatures Associated With Vertebrate Viviparity.” BMC Biology 22, no. 1: 34. 10.1186/S12915-024-01837-W. 38331819 PMC10854053

[mec70469-bib-0024] Fagerberg, L. , B. M. Hallstrom , P. Oksvold , et al. 2014. “Analysis of the Human Tissue‐Specific Expression by Genome‐Wide Integration of Transcriptomics and Antibody‐Based Proteomics.” Molecular & Cellular Proteomics 13, no. 2: 397–406. 10.1074/MCP.M113.035600. 24309898 PMC3916642

[mec70469-bib-0025] Foster, C. S. P. , M. B. Thompson , J. U. Van Dyke , M. C. Brandley , and C. M. Whittington . 2020. “Emergence of an Evolutionary Innovation: Gene Expression Differences Associated With the Transition Between Oviparity and Viviparity.” Molecular Ecology 29, no. 7: 1315–1327. 10.1111/mec.15409. 32153075

[mec70469-bib-0026] Foster, C. S. P. , J. U. Van Dyke , M. B. Thompson , et al. 2022. “Different Genes Are Recruited During Convergent Evolution of Pregnancy and the Placenta.” Molecular Biology and Evolution 39, no. 4. 10.1093/MOLBEV/MSAC077. PMC9048886 35388432

[mec70469-bib-0027] Gao, C. H. , C. Chen , T. Akyol , et al. 2024. “ggVennDiagram: Intuitive Venn Diagram Software Extended.” iMeta 3, no. 1: e177. 10.1002/IMT2.177. 38868514 PMC10989133

[mec70469-bib-0028] Gao, H. , G. Wu , T. E. Spencer , G. A. Johnson , and F. W. Bazer . 2009. “Select Nutrients in the Ovine Uterine Lumen. V. Nitric Oxide Synthase, GTP Cyclohydrolase, and Ornithine Decarboxylase in Ovine Uteri and Peri‐Implantation Conceptuses.” Biology of Reproduction 81, no. 1: 67–76. 10.1095/BIOLREPROD.108.075473. 19246319

[mec70469-bib-0029] Gao, W. , Y. B. Sun , W. W. Zhou , et al. 2019. “Genomic and Transcriptomic Investigations of the Evolutionary Transition From Oviparity to Viviparity.” Proceedings of the National Academy of Sciences of the United States of America 116, no. 9: 3646–3655. 10.1073/pnas.1816086116. 30808754 PMC6397529

[mec70469-bib-0030] Gerlevik, S. , N. Seymen , S. Hama , et al. 2024. “Identification of Novel Myelodysplastic Syndromes Prognostic Subgroups by Integration of Inflammation, Cell‐Type Composition, and Immune Signatures in the Bone Marrow.” eLife 13. 10.7554/ELIFE.97096. PMC11377035 39235452

[mec70469-bib-0031] Girotti, M. , and H. H. Zingg . 2003. “Gene Expression Profiling of Rat Uterus at Different Stages of Parturition.” Endocrinology 144, no. 6: 2254–2265. 10.1210/EN.2002-0196. 12746283

[mec70469-bib-0032] GitHub . n.d. rrwick/Filtlong: Quality Filtering Tool for Long Reads. https://github.com/rrwick/Filtlong.

[mec70469-bib-0033] Gopalakrishnan, K. , and S. Kumar . 2020. “Whole‐Genome Uterine Artery Transcriptome Profiling and Alternative Splicing Analysis in Rat Pregnancy.” International Journal of Molecular Sciences 21, no. 6: 2079. 10.3390/IJMS21062079. 32197362 PMC7139363

[mec70469-bib-0034] Graham, S. P. , R. L. Earley , C. Guyer , and M. T. Mendonça . 2011. “Innate Immune Performance and Steroid Hormone Profiles of Pregnant Versus Nonpregnant Cottonmouth Snakes ( *Agkistrodon piscivorus* ).” General and Comparative Endocrinology 174, no. 3: 348–353. 10.1016/J.YGCEN.2011.09.015. 21986088

[mec70469-bib-0035] Griffith, O. W. , M. C. Brandley , K. Belov , and M. B. Thompson . 2016. “Reptile Pregnancy Is Underpinned by Complex Changes in Uterine Gene Expression: A Comparative Analysis of the Uterine Transcriptome in Viviparous and Oviparous Lizards.” Genome Biology and Evolution 8, no. 10: 3226–3239. 10.1093/gbe/evw229. 27635053 PMC5174741

[mec70469-bib-0036] Griffith, O. W. , A. R. Chavan , S. Protopapas , J. Maziarz , R. Romero , and G. P. Wagner . 2017. “Embryo Implantation Evolved From an Ancestral Inflammatory Attachment Reaction.” Proceedings of the National Academy of Sciences of the United States of America 114, no. 32. 10.1073/pnas.1701129114. PMC5559003 28747528

[mec70469-bib-0037] Gu, Y. , Y. He , X. Zhang , et al. 2015. “Deficiency of Monoclonal Non‐Specific Suppressor Factor Beta (MNSFB) Promotes Pregnancy Loss in Mice.” Molecular Reproduction and Development 82, no. 6: 475–488. 10.1002/MRD.22495. 26031240

[mec70469-bib-0038] Gu, Y. , J. M. Wang , Z. F. Zhang , et al. 2018. “The Association Between Polymorphisms of Genes Related to Inflammation and Recurrent Pregnancy Loss.” Gynecological Endocrinology 34, no. 4: 349–352. 10.1080/09513590.2017.1395837. 29084471

[mec70469-bib-0039] Guillette, L. J. 1993. “The Evolultion of Viviparity in Lizards. Ecological, Anatomical, and Physiological Correlates Lead to New Hypotheses.” Bioscience 43, no. 11: 742–751. 10.2307/1312318.

[mec70469-bib-0040] Hallgren, J. , K. D. Tsirigos , M. Damgaard Pedersen , et al. 2022. “DeepTMHMM Predicts Alpha and Beta Transmembrane Proteins Using Deep Neural Networks.” *bioRxiv*. 10.1101/2022.04.08.487609.

[mec70469-bib-0041] Hansen, M. D. H. , and M. C. Beckerle . 2006. “Opposing Roles of Zyxin/LPP ACTA Repeats and the LIM Domain Region in Cell‐Cell Adhesion.” Journal of Biological Chemistry 281, no. 23: 16178–16188. 10.1074/jbc.M512771200. 16613855

[mec70469-bib-0042] Hansen, M. D. H. , and M. C. Beckerle . 2008. “α‐Actinin Links LPP, but Not Zyxin, to Cadherin‐Based Junctions.” Biochemical and Biophysical Research Communications 371, no. 1: 144–148. 10.1016/J.BBRC.2008.04.018. 18413140 PMC2676570

[mec70469-bib-0043] Hansen, V. L. , F. D. Schilkey , and R. D. Miller . 2016. “Transcriptomic Changes Associated With Pregnancy in a Marsupial, the Gray Short‐Tailed Opossum *Monodelphis domestica* .” PLoS One 11, no. 9: 1–25. 10.1371/journal.pone.0161608. PMC5012577 27598793

[mec70469-bib-0044] Hendrawan, K. , C. M. Whittington , M. C. Brandley , K. Belov , and M. B. Thompson . 2017. “The Regulation of Uterine Proinflammatory Gene Expression During Pregnancy in the Live‐Bearing Lizard, *Pseudemoia entrecasteauxii* .” Journal of Experimental Zoology. Part B, Molecular and Developmental Evolution 328, no. 4: 334–346. 10.1002/JEZ.B.22733. 28296138

[mec70469-bib-0045] Hoffmann, F. G. , M. W. Vandewege , J. F. Storz , and J. C. Opazo . 2018. “Gene Turnover and Diversification of the α‐ and β‐Globin Gene Families in Sauropsid Vertebrates.” Genome Biology and Evolution 10, no. 1: 344–358. 10.1093/GBE/EVY001. 29340581 PMC5786229

[mec70469-bib-0046] Home Office . 2013. “Animals (Scientific Procedures) Act 1986 Amendment Regulations 2012.” https://www.gov.uk/government/uploads/system/uploads/attachment_data/file/619140/ConsolidatedASPA1Jan2013.pdf.

[mec70469-bib-0048] Huerta‐Cepas, J. , D. Szklarczyk , D. Heller , et al. 2019. “eggNOG 5.0: A Hierarchical, Functionally and Phylogenetically Annotated Orthology Resource Based on 5090 Organisms and 2502 Viruses.” Nucleic Acids Research 47, no. D1: D309–D314. 10.1093/nar/gky1085. 30418610 PMC6324079

[mec70469-bib-0049] Jacobs, A. , and K. R. Elmer . 2020. “Alternative Splicing and Gene Expression Play Contrasting Roles in the Parallel Phenotypic Evolution of a Salmonid Fish.” *bioRxiv*. 10.1101/2020.05.11.087973. PMC8653899 33502030

[mec70469-bib-0050] Jin, Y. , S. Yang , X. Gao , et al. 2022. “DEAD‐Box Helicase 27 Triggers Epithelial to Mesenchymal Transition by Regulating Alternative Splicing of Lipoma‐Preferred Partner in Gastric Cancer Metastasis.” Frontiers in Genetics 13: 836199. 10.3389/FGENE.2022.836199/BIBTEX. 35601484 PMC9114675

[mec70469-bib-0051] Kas, K. , L. Michiels , and J. Merregaert . 1992. “Genomic Structure and Expression of the Human Fau Gene: Encoding the Ribosomal Protein S30 Fused to a Ubiquitin‐Like Protein.” Biochemical and Biophysical Research Communications 187, no. 2: 927–933. 10.1016/0006-291X(92)91286-Y. 1326960

[mec70469-bib-0052] Khorram, O. , M. Garthwaite , and R. R. Magness . 1999. “Endometrial and Myometrial Expression of Nitric Oxide Synthase Isoforms in Pre‐ and Postmenopausal Women.” Journal of Clinical Endocrinology and Metabolism 84, no. 6: 2226–2232. 10.1210/JCEM.84.6.5759. 10372735

[mec70469-bib-0053] Kidron, T. , P. F. Kraicer , S. Polak‐Charcon , A. Amit , J. B. Lessing , and U. Barkai . 1995. “Excess Expression of Uterine Ribosomal Protein Genes P2 and S25 During the ‘Implantation Window’ in the Rat.” 2, no. 5: 700–707. 10.1177/107155769500200506. 9420878

[mec70469-bib-0054] Komeno, Y. , J. Qiu , L. Lin , et al. 2013. “SRSF2 Is Essential for Hematopoiesis and Its Mutations Dysregulate Alternative RNA Splicing in MDS.” Blood 122, no. 21: 261. 10.1182/BLOOD.V122.21.261.261.

[mec70469-bib-0055] Kramer, A. 1996. “The Structure and Function of Proteins Involved in Mammalian Pre‐mRNA Splicing.” Annual Review of Biochemistry 65: 367–409. https://www.annualreviews.org. 10.1146/annurev.bi.65.070196.002055 8811184

[mec70469-bib-0056] Kravitz, R. H. , R. L. Grendell , I. I. Slukvin , and T. G. Golos . 2001. “Selective Expression of NKG2‐A and NKG2‐C mRNAs and Novel Alternative Splicing of 5′ Exons in Rhesus Monkey Decidua.” Immunogenetics 53, no. 1: 69–73. 10.1007/S002510000289/METRICS. 11261935

[mec70469-bib-0057] Lawrence, M. , W. Huber , H. Pagès , et al. 2013. “Software for Computing and Annotating Genomic Ranges.” PLoS Computational Biology 9, no. 8: e1003118. 10.1371/JOURNAL.PCBI.1003118. 23950696 PMC3738458

[mec70469-bib-0058] Lei, W. L. , Z. Du , T. G. Meng , et al. 2023. “SRSF2 Is Required for mRNA Splicing During Spermatogenesis.” BMC Biology 21, no. 1: 1–15. 10.1186/S12915-023-01736-6/FIGURES/7. 37867192 PMC10591377

[mec70469-bib-0059] Li, H. 2018. “Minimap2: Pairwise Alignment for Nucleotide Sequences.” Bioinformatics 34, no. 18: 3094–3100. 10.1093/BIOINFORMATICS/BTY191. 29750242 PMC6137996

[mec70469-bib-0060] Lin, Z. , and W. H. Li . 2012. “Evolution of 5′ Untranslated Region Length and Gene Expression Reprogramming in Yeasts.” Molecular Biology and Evolution 29, no. 1: 81–89. 10.1093/MOLBEV/MSR143. 21965341 PMC3245540

[mec70469-bib-0061] Linville, B. J. , J. R. Stewart , T. W. Ecay , J. F. Herbert , S. L. Parker , and M. B. Thompson . 2010. “Placental Calcium Provision in a Lizard With Prolonged Oviductal Egg Retention.” Journal of Comparative Physiology B Biochemical, Systemic, and Environmental Physiology 180, no. 2: 221–227. 10.1007/S00360-009-0400-2/TABLES/4. 19727762

[mec70469-bib-0062] Liu, X. , L. Zhang , J. Han , et al. 2020. “A Comparative Analysis of Gene Expression Induced by the Embryo in the Caprine Endometrium.” Veterinary Medicine and Science 6, no. 2: 196. 10.1002/VMS3.221. 31782264 PMC7196676

[mec70469-bib-0063] Lourdais, O. , S. Lorioux , A. Dupoué , C. Wright , and D. F. DeNardo . 2015. “Embryonic Water Uptake During Pregnancy Is Stage‐ and Fecundity‐Dependent in the Snake *Vipera aspis* .” Comparative Biochemistry and Physiology Part A: Molecular & Integrative Physiology 189: 102–106. 10.1016/J.CBPA.2015.07.019. 26255703

[mec70469-bib-0064] Love, M. I. , W. Huber , and S. Anders . 2014. “Moderated Estimation of Fold Change and Dispersion for RNA‐Seq Data With DESeq2.” Genome Biology 15, no. 12: 550. 10.1186/S13059-014-0550-8/FIGURES/9. 25516281 PMC4302049

[mec70469-bib-0065] Love, M. I. , C. Soneson , and R. Patro . 2018. “Swimming Downstream: Statistical Analysis of Differential Transcript Usage Following Salmon Quantification.” F1000Research 7: 952. 10.12688/f1000research.15398.3. 30356428 PMC6178912

[mec70469-bib-0066] Ma, X.‐H. , S.‐J. Hu , H. Ni , et al. 2006. “Serial Analysis of Gene Expression in Mouse Uterus at the Implantation Site.” Journal of Biological Chemistry 281: 9351–9360. 10.1074/jbc.M511512200. 16434403

[mec70469-bib-0067] Mathies, T. , and R. M. Andrews . 1996. “Extended Egg Retention and Its Influence on Embryonic Development and Egg Water Balance.” Implications for the Evolution of Viviparity 69, no. 5: 1021–1035. 10.1086/PHYSZOOL.69.5.30164244.

[mec70469-bib-0068] McGowen, M. R. , O. Erez , R. Romero , and D. E. Wildman . 2014. “The Evolution of Embryo Implantation.” International Journal of Developmental Biology 58, no. 2–4: 155. 10.1387/IJDB.140020DW. 25023681 PMC6053685

[mec70469-bib-0069] Merkl, M. , S. E. Ulbrich , C. Otzdorff , et al. 2010. “Microarray Analysis of Equine Endometrium at Days 8 and 12 of Pregnancy.” Biology of Reproduction 83, no. 5: 874–886. 10.1095/BIOLREPROD.110.085233. 20631402

[mec70469-bib-0070] Mika, K. , C. M. Whittington , B. M. McAllan , and V. J. Lynch . 2022. “Gene Expression Phylogenies and Ancestral Transcriptome Reconstruction Resolves Major Transitions in the Origins of Pregnancy.” eLife 11. 10.7554/ELIFE.74297. PMC9275820 35770963

[mec70469-bib-0071] Mistry, J. , S. Chuguransky , L. Williams , et al. 2021. “Pfam: The Protein Families Database in 2021.” Nucleic Acids Research 49, no. D1: D412–D419. 10.1093/NAR/GKAA913. 33125078 PMC7779014

[mec70469-bib-0072] Money, T. T. , R. G. King , M. H. Wong , et al. 2007. “Expression and Cellular Localisation of Chloride Intracellular Channel 3 in Human Placenta and Fetal Membranes.” Placenta 28, no. 5–6: 429–436. 10.1016/j.placenta.2006.08.002. 17027078

[mec70469-bib-0073] Murthi, P. , J. L. Stevenson , T. T. Money , A. J. Borg , S. P. Brennecke , and N. M. Gude . 2012. “Placental CLIC3 Is Increased in Fetal Growth Restriction and Pre‐Eclampsia Affected Human Pregnancies.” Placenta 33, no. 9: 741–744. 10.1016/J.PLACENTA.2012.06.011. 22795578

[mec70469-bib-0074] Nakamura, M. , H. Ogawa , and T. Tsunematsu . 1987. “Characterization of Monoclonal Nonspecific Suppressor Factor (MNSF) With the Use of a Monoclonal Antibody.” Journal of Immunology 138, no. 6: 1799–1803. 10.4049/JIMMUNOL.138.6.1799. 3102600

[mec70469-bib-0075] Nakamura, M. , and N. Watanabe . 2010. “Ubiquitin‐Like Protein MNSFβ/Endophilin II Complex Regulates Dectin‐1‐Mediated Phagocytosis and Inflammatory Responses in Macrophages.” Biochemical and Biophysical Research Communications 401, no. 2: 257–261. 10.1016/J.BBRC.2010.09.045. 20849826

[mec70469-bib-0076] Nie, G. Y. , A. L. Hampton , G. Q. Fu , Y. X. Liu , J. K. Findlay , and L. A. Salamonsen . 2002. “A Potential Molecular Mechanism for Regulating Pre‐mRNA Splicing of Implantation‐Related Genes Through Unique Uterine Expression of Splicing Factor SC35 in Women and Rhesus Monkeys.” Reproduction 124, no. 2: 209–217. 10.1530/REP.0.1240209. 12141933

[mec70469-bib-0077] Nie, G. Y. , Y. Li , L. Batten , et al. 2000. “Uterine Expression of Alternatively Spliced mRNAs of Mouse Splicing Factor SC35 During Early Pregnancy.” Molecular Human Reproduction 6, no. 12: 1131–1139. 10.1093/MOLEHR/6.12.1131. 11101696

[mec70469-bib-0078] Nie, G. Y. , Y. Li , A. L. Hampton , L. A. Salamonsen , J. A. Clements , and J. K. Findlay . 2000. “Identification of Monoclonal Nonspecific Suppressor Factor Beta (MNSF) as One of the Genes Differentially Expressed at Implantation Sites Compared to Interimplantation Sites in the Mouse Uterus.” Molecular Reproduction and Development 55: 351–363. 10.1002/(SICI)1098-2795(200004)55:4. 10694741

[mec70469-bib-0079] Pan, Q. , O. Shai , L. J. Lee , B. J. Frey , and B. J. Blencowe . 2008. “Deep Surveying of Alternative Splicing Complexity in the Human Transcriptome by High‐Throughput Sequencing.” Nature Genetics 40, no. 12: 1413–1415. 10.1038/ng.259. 18978789

[mec70469-bib-0080] Patro, R. , G. Duggal , M. I. Love , R. A. Irizarry , and C. Kingsford . 2017. “Salmon: Fast and Bias‐Aware Quantification of Transcript Expression Using Dual‐Phase Inference.” Nature Methods 14, no. 4: 417. 10.1038/NMETH.4197. 28263959 PMC5600148

[mec70469-bib-0081] Petit, M. M. R. , J. Fradelizi , R. M. Golsteyn , et al. 2000. “LPP, an Actin Cytoskeleton Protein Related to Zyxin, Harbors a Nuclear Export Signal and Transcriptional Activation Capacity.” Molecular Biology of the Cell 11: 117–129. 10637295 10.1091/mbc.11.1.117 PMC14761

[mec70469-bib-0082] Petit, M. M. R. , R. Mols , E. F. P. M. Schoenmakers , N. Mandahl , and W. J. M. Van De Ven . 1996. “LPP,the Preferred Fusion Partner Gene ofHMGICin Lipomas, Is a Novel Member of the LIM Protein Gene Family.” Genomics 36, no. 1: 118–129. 10.1006/GENO.1996.0432. 8812423

[mec70469-bib-0083] Pollyea, D. A. , C. Harris , J. L. Rabe , et al. 2019. “Myelodysplastic Syndrome‐Associated Spliceosome Gene Mutations Enhance Innate Immune Signaling.” Haematologica 104, no. 9: e388–e392. 10.3324/HAEMATOL.2018.214155. 30846499 PMC6717580

[mec70469-bib-0084] R Core Team . 2023. “R: A Language and Environment for Statistical Computing.” https://www.R‐project.org/.

[mec70469-bib-0085] Ran, Y. , J. He , R. Chen , et al. 2022. “Investigation and Validation of Molecular Characteristics of Endometrium in Recurrent Miscarriage and Unexplained Infertility From a Transcriptomic Perspective.” International Journal of Medical Sciences 19, no. 3: 546. 10.7150/IJMS.69648. 35370464 PMC8964333

[mec70469-bib-0086] Recknagel, H. , M. Carruthers , A. A. Yurchenko , et al. 2021. “The Functional Genetic Architecture of Egg‐Laying and Live‐Bearing Reproduction in Common Lizards.” Nature Ecology & Evolution 5, no. 11: 1546–1556. 10.1038/s41559-021-01555-4. 34621056

[mec70469-bib-0087] Reijnders, M. J. M. F. , and R. M. Waterhouse . 2021. “Summary Visualizations of Gene Ontology Terms With GO‐Figure!” Frontiers in Bioinformatics 1: 638255. 10.3389/FBINF.2021.638255/BIBTEX. 36303779 PMC9581009

[mec70469-bib-0088] Resch, A. , Y. Xing , B. Modrek , M. Gorlick , R. Riley , and C. Lee . 2004. “Assessing the Impact of Alternative Splicing on Domain Interactions in the Human Proteome.” Journal of Proteome Research 3, no. 1: 76–83. 10.1021/PR034064V. 14998166

[mec70469-bib-0089] Robinson, M. D. , and M. Nowicka . 2016. “DRIMSeq: A Dirichlet‐Multinomial Framework for Multivariate Count Outcomes in Genomics.” F1000Research 5. 10.12688/F1000RESEARCH.8900.2. PMC5200948 28105305

[mec70469-bib-0090] Rogers, T. F. , D. H. Palmer , and A. E. Wright . 2021. “Sex‐Specific Selection Drives the Evolution of Alternative Splicing in Birds.” Molecular Biology and Evolution 38, no. 2: 519–530. 10.1093/MOLBEV/MSAA242. 32977339 PMC7826194

[mec70469-bib-0091] Ruano, C. S. M. , C. Apicella , S. Jacques , et al. 2021. “Alternative Splicing in Normal and Pathological Human Placentas Is Correlated to Genetic Variants.” Human Genetics 140, no. 5: 827–848. 10.1007/S00439-020-02248-X/FIGURES/7. 33433680 PMC8052246

[mec70469-bib-0092] Ryczek, N. , A. Łyś , and I. Makałowska . 2023. “The Functional Meaning of 5′ UTR in Protein‐Coding Genes.” International Journal of Molecular Sciences 24, no. 3: 2976. 10.3390/IJMS24032976. 36769304 PMC9917990

[mec70469-bib-0093] Saad, A. H. , and S. El Deeb . 1990. “Immunological Changes During Pregnancy in the Viviparous Lizard, *Chalcides ocellatus* .” Veterinary Immunology and Immunopathology 25, no. 3: 279–286. 10.1016/0165-2427(90)90051-S. 2396365

[mec70469-bib-0094] Salamonsen, L. A. , G. Nie , and J. K. Findlay . 2002. “Newly Identified Endometrial Genes of Importance for Implantation.” Journal of Reproductive Immunology 53, no. 1–2: 215–225. 10.1016/S0165-0378(01)00087-0. 11730918

[mec70469-bib-0095] Samborski, A. , A. Graf , S. Krebs , et al. 2013. “Transcriptome Changes in the Porcine Endometrium During the Preattachment Phase.” Biology of Reproduction 89, no. 6. 10.1095/BIOLREPROD.113.112177. 24174570

[mec70469-bib-0096] Sartori, M. R. , A. S. Abe , D. A. Crossley , and E. W. Taylor . 2017. “Rates of Oxygen Uptake Increase Independently of Changes in Heart Rate in Late Stages of Development and at Hatching in the Green Iguana, *Iguana iguana* .” Comparative Biochemistry and Physiology Part A: Molecular & Integrative Physiology 205: 28–34. 10.1016/J.CBPA.2016.12.020. 28011410

[mec70469-bib-0097] Shine, R. , and L. J. Guillette . 1988. “The Evolution of Viviparity in Reptiles: A Physiological Model and Its Ecological Consequences.” Journal of Theoretical Biology 132, no. 1: 43–50. 10.1016/S0022-5193(88)80189-9.

[mec70469-bib-0098] Singh, P. , and E. P. Ahi . 2022. “The Importance of Alternative Splicing in Adaptive Evolution.” Molecular Ecology 31, no. 7. 10.1111/mec.16377. 35094439

[mec70469-bib-0099] Smout, J. L. , M. M. Bain , M. McLaughlin , and K. R. Elmer . 2024. “Common Lizard Primary Oviduct Cell Culture: A Model System for the Genetic and Cellular Basis of Oviparity and Viviparity.” Experimental Cell Research 442, no. 1: 114196. 10.1016/J.YEXCR.2024.114196. 39117090

[mec70469-bib-0100] Soergel, D. A. W. , L. F. Lareau , and S. E. Brenner . 2013. “Regulation of Gene Expression by Coupling of Alternative Splicing and NMD.” https://www.ncbi.nlm.nih.gov/books/NBK6088/.

[mec70469-bib-0101] Soneson, C. , M. I. Love , and M. D. Robinson . 2015. “Differential Analyses for RNA‐Seq: Transcript‐Level Estimates Improve Gene‐Level Inferences [Version 1; Referees: 2 Approved].” 10.12688/f1000research.7563.1. PMC4712774 26925227

[mec70469-bib-0102] Stewart, J. R. 2013. “Fetal Nutrition in Lecithotrophic Squamate Reptiles: Toward a Comprehensive Model for Evolution of Viviparity and Placentation.” Journal of Morphology 274, no. 7: 824–843. 10.1002/JMOR.20141. 23520054

[mec70469-bib-0103] Stewart, J. R. , T. W. Ecay , C. P. Garland , et al. 2009. “Maternal Provision and Embryonic Uptake of Calcium in an Oviparous and a Placentotrophic Viviparous Australian Lizard (Lacertilia: Scincidae).” Comparative Biochemistry and Physiology Part A: Molecular & Integrative Physiology 153, no. 2: 202–208. 10.1016/J.CBPA.2009.02.014. 19223019

[mec70469-bib-0104] Stewart, J. R. , B. Heulin , and Y. Surget‐Groba . 2004. “Extraembryonic Membrane Development in a Reproductively Bimodal Lizard, Lacerta (Zootoca) Vivipara.” Zoology 107, no. 4: 289–314. 10.1016/J.ZOOL.2004.07.004. 16351946

[mec70469-bib-0105] Sudhir, N. , B. A. Badaruddoza , and A. Kaur . 2016. “Association of Tumor Necrosis Factor‐Alpha 308G/A Polymorphism With Recurrent Miscarriages in Women.” Journal of Human Reproductive Sciences 9, no. 2: 86. 10.4103/0974-1208.183516. 27382232 PMC4915291

[mec70469-bib-0106] Sureau, A. , and B. Perbal . 1994. “Several mRNAs With Variable 3′ Untranslated Regions and Different Stability Encode the Human PR264/SC35 Splicing Factor.” Proceedings of the National Academy of Sciences of the United States of America 91, no. 3: 932–936. 10.1073/PNAS.91.3.932. 8302870 PMC521427

[mec70469-bib-0107] Teufel, F. , J. J. Almagro Armenteros , A. R. Johansen , et al. 2022. “SignalP 6.0 Predicts All Five Types of Signal Peptides Using Protein Language Models.” Nature Biotechnology 40, no. 7: 1023–1025. 10.1038/s41587-021-01156-3. PMC9287161 34980915

[mec70469-bib-0108] Thumuluri, V. , J. J. Almagro Armenteros , A. R. Johansen , H. Nielsen , and O. Winther . 2022. “DeepLoc 2.0: Multi‐Label Subcellular Localization Prediction Using Protein Language Models.” Nucleic Acids Research 50, no. W1: W228–W234. 10.1093/NAR/GKAC278. 35489069 PMC9252801

[mec70469-bib-0109] Van den Berge, K. , C. Soneson , M. D. Robinson , and L. Clement . 2017. “stageR: A General Stage‐Wise Method for Controlling the Gene‐Level False Discovery Rate in Differential Expression and Differential Transcript Usage.” Genome Biology 18, no. 1: 1–14. 10.1186/S13059-017-1277-0. 28784146 PMC5547545

[mec70469-bib-0110] van den Heuvel, J. , C. Ashiono , L. Gillet , et al. 2021. “Processing of the Ribosomal Ubiquitin‐Like Fusion Protein FUBI‐eS30/FAU Is Required for 40S Maturation and Depends on USP36.” eLife 10. 10.7554/ELIFE.70560. PMC8354635 34318747

[mec70469-bib-0111] Van Der Velden, A. W. , and A. A. M. Thomas . 1999. “The Role of the 5′ Untranslated Region of an mRNA in Translation Regulation During Development.” International Journal of Biochemistry & Cell Biology 31, no. 1: 87–106. 10.1016/S1357-2725(98)00134-4. 10216946

[mec70469-bib-0112] Van Dyke, J. U. , M. C. Brandley , and M. B. Thompson . 2014. “The Evolution of Viviparity: Molecular and Genomic Data From Squamate Reptiles Advance Understanding of Live Birth in Amniotes.” Reproduction 147, no. 1: R15–R26. 10.1530/REP-13-0309. 24129151

[mec70469-bib-0113] Verta, J. P. , and A. Jacobs . 2022. “The Role of Alternative Splicing in Adaptation and Evolution.” Trends in Ecology & Evolution 37, no. 4: 299–308. 10.1016/J.TREE.2021.11.010. 34920907

[mec70469-bib-0114] Vervenne, H. B. V. K. , K. R. M. O. Crombez , E. L. Delvaux , V. Janssens , W. J. M. Van de Ven , and M. M. R. Petit . 2009. “Targeted Disruption of the Mouse Lipoma Preferred Partner Gene.” Biochemical and Biophysical Research Communications 379, no. 2: 368–373. 10.1016/J.BBRC.2008.12.074. 19111675

[mec70469-bib-0115] Vinjamur, D. S. , D. E. Bauer , and S. H. Orkin . 2018. “Recent Progress in Understanding and Manipulating Haemoglobin Switching for the Haemoglobinopathies.” British Journal of Haematology 180, no. 5: 630–643. 10.1111/BJH.15038. 29193029

[mec70469-bib-0116] Vitting‐Seerup, K. , A. Sandelin , and B. Berger . 2019. “IsoformSwitchAnalyzeR: Analysis of Changes in Genome‐Wide Patterns of Alternative Splicing and Its Functional Consequences.” Bioinformatics 35, no. 21: 4469–4471. 10.1093/BIOINFORMATICS/BTZ247. 30989184

[mec70469-bib-0117] Wang, H. Y. , X. Xu , J. H. Ding , J. R. Bermingham , and X. D. Fu . 2001. “SC35 Plays a Role in T Cell Development and Alternative Splicing of CD45.” Molecular Cell 7, no. 2: 331–342. 10.1016/S1097-2765(01)00181-2. 11239462

[mec70469-bib-0118] Welter, H. , H. Bollwein , F. Weber , S. Rohr , and R. Einspanier . 2005. “Expression of Endothelial and Inducible Nitric Oxide Synthases Is Modulated in the Endometrium of Cyclic and Early Pregnant Mares.” Reproduction, Fertility and Development 16, no. 7: 689–698. 10.1071/RD03103. 15740692

[mec70469-bib-0119] Whittington, C. M. , M. J. Hodgson , and C. R. Friesen . 2025. “Convergent Evolution of Pregnancy in Vertebrates.” Annual Review of Animal Biosciences 13, no. 1: 189–209. 10.1146/ANNUREV-ANIMAL-111523-102029/CITE/REFWORKS. 39546412

[mec70469-bib-0120] Whittington, C. M. , J. U. Van Dyke , S. Q. T. Liang , et al. 2022. “Understanding the Evolution of Viviparity Using Intraspecific Variation in Reproductive Mode and Transitional Forms of Pregnancy.” Biological Reviews of the Cambridge Philosophical Society 97, no. 3: 1179. 10.1111/BRV.12836. 35098647 PMC9064913

[mec70469-bib-0121] Wick, R. R. , L. M. Judd , C. L. Gorrie , and K. E. Holt . 2017. “Completing Bacterial Genome Assemblies With Multiplex MinION Sequencing.” Microbial Genomics 3, no. 10. 10.1099/MGEN.0.000132. PMC5695209 29177090

[mec70469-bib-0122] Wick, R. R. , L. M. Judd , and K. E. Holt . 2019. “Performance of Neural Network Basecalling Tools for Oxford Nanopore Sequencing.” Genome Biology 20, no. 1: 1–10. 10.1186/S13059-019-1727-Y/FIGURES/4. 31234903 PMC6591954

[mec70469-bib-0123] Wickham, H. 2009. “ggplot2. Ggplot2.” 10.1007/978-0-387-98141-3.

[mec70469-bib-0124] Wieder, N. , E. N. D'Souza , A. C. Martin‐Geary , et al. 2024. “Differences in 5′ Untranslated Regions Highlight the Importance of Translational Regulation of Dosage Sensitive Genes.” Genome Biology 25, no. 1: 1–16. 10.1186/S13059-024-03248-0/FIGURES/4. 38685090 PMC11057154

[mec70469-bib-0125] Xie, H. , D. Hunter , J. L. Smout , O. E. Gaggiotti , and K. R. Elmer . 2025. “Convergent Molecular Evolution of Viviparity Across Squamate Reptiles.” *bioRxiv*. 10.1101/2025.09.26.678543.

[mec70469-bib-0126] Xue, L. L. , F. Wang , L. L. Xiong , et al. 2020. “A Single‐Nucleotide Polymorphism Induced Alternative Splicing in Tacr3 Involves in Hypoxic‐Ischemic Brain Damage.” Brain Research Bulletin 154: 106–115. 10.1016/J.BRAINRESBULL.2019.11.001. 31722250

[mec70469-bib-0127] Yang, P. , D. Wang , and L. Kang . 2021. “Alternative Splicing Level Related to Intron Size and Organism Complexity.” BMC Genomics 22, no. 1: 1–16. 10.1186/S12864-021-08172-2/FIGURES/6. 34819032 PMC8614042

[mec70469-bib-0128] Yatsenko, O. P. , M. L. Filipenko , E. A. Khrapov , E. N. Voronina , S. V. Sennikov , and V. A. Kozlov . 2004. “Alternative Splicing of Interleukin‐6 mRNA in Mice.” Bulletin of Experimental Biology and Medicine 138, no. 7: 73–76. 10.1007/BF02694480. 15514729

[mec70469-bib-0129] Yoshiki, N. , T. Kubota , and T. Aso . 2000. “Expression and Localization of Inducible Nitric Oxide Synthase in Human Non‐Pregnant and Early Pregnant Endometrium.” Molecular Human Reproduction 6, no. 3: 283–287. 10.1093/MOLEHR/6.3.283. 10694278

[mec70469-bib-0130] Yusuf, L. H. , Y. S. Lemus , P. Thorpe , C. M. Garcia , and M. G. Ritchie . 2023. “Genomic Signatures Associated With Transitions to Viviparity in Cyprinodontiformes.” Molecular Biology and Evolution 40, no. 10. 10.1093/MOLBEV/MSAD208. PMC10568250 37789509

[mec70469-bib-0131] Zatta, S. , H. Rehrauer , A. Gram , A. Boos , and M. P. Kowalewski . 2017. “Transcriptome Analysis Reveals Differences in Mechanisms Regulating Cessation of Luteal Function in Pregnant and Non‐Pregnant Dogs.” BMC Genomics 18, no. 1: 1–18. 10.1186/S12864-017-4084-9/FIGURES/8. 28954628 PMC5618722

[mec70469-bib-0132] Zeng, W. , X. Liu , Z. Liu , et al. 2018. “Deep Surveying of the Transcriptional and Alternative Splicing Signatures for Decidual CD8+ T Cells at the First Trimester of Human Healthy Pregnancy.” Frontiers in Immunology 9: 342710. 10.3389/FIMMU.2018.00937/BIBTEX. PMC5946033 29780389

