# Supplementary material for: Gene Expression and Alternative Splicing Throughout the Reproductive Cycle of a Viviparous Lizard Reveal Novel Genes for Pregnancy and Convergence With Squamates and Mammals
Source: Mol Ecol. 2026 Jul 21;35(14):e70469. doi: 10.1111/mec.70469 (PMC13387830; doi:10.1111/mec.70469)
Supplement: Supplementary file 1 — Supplementary Results: Differential gene expression sensitivity analysis, Differential transcript usage sensitivity analysis. Figure S1: Differential gene expression before and after pregnancy in Z. vivipara. Figure S2: Comparison of differential gene expression (DGE) and differential transcript usage (DTU) during pregnancy in Z. vivipara. Figure S3: Boxplots showing expression levels (normalised read counts) of two candidate genes differentially expressed during pregnancy in the Z. vivipara uterus. Figure S4: Significant DEGs when subsampling pregnant uterus samples to equalise sample size. Figure S5: Genes with evidence of significant differential transcript usage when subsampling pregnant uterus samples to equalise sample size. Figure S6: Principal component analysis (PCA) of transcript‐level read counts after filtering and normalisation. [file MEC-35-e70469-s001.docx]

**Supplementary Material for**

Gene expression and alternative splicing throughout the reproductive cycle of a viviparous lizard reveal novel genes for pregnancy and convergence with squamates and mammals

John L Smout, Maureen M Bain, Mark McLaughlin, Kathryn R Elmer

**Supplementary Tables**

Supplementary Tables 1-21 and legends in excel file

**Supplementary results**

# Differential gene expression sensitivity analysis

To evaluate the robustness of our differential gene expression analysis to unequal sample size (n = 4 for pregnancy vs n = 2 for pre-pregnancy and n = 2 post-parturition) we repeated our differential gene expression analysis with DESeq2 using the same settings but subsampling down to two pregnancy samples (for all six possible permutations). We compared these results to the full n = 4 DGE results in terms of log FC and the resulting set of significant genes (p-adj < 0.1, absolute log FC > 1).

Per-gene log FC values were highly correlated for all six permutations when comparing pregnancy either to pre-pregnancy (mean Spearman’s ρ = 0.9515) or post-parturition (mean Spearman’s ρ = 0.9507) (**Table S20**). We compared the lists of DEGs obtained for each permutation and for the full analysis and found that the largest intersection was DEGs shared between all permutations, both for pre-pregnancy (235 genes, **Fig. S4A**) and post-parturition (180 genes, **Fig. S4B**), with the second largest group being genes unique to the full analysis (i.e. detected as DEGs when all four samples are considered, but not when subsampling to any two individual samples). This is expected, because the power of the analysis to detect differentially expressed genes increases with the addition of more samples. These results give us high confidence in the robustness of our full analysis despite the difference in sample size.

# Differential transcript usage sensitivity analysis

To evaluate the robustness of our differential transcript usage analysis to unequal sample size (n = 4 for pregnancy vs n = 2 for pre-pregnancy and n = 2 post-parturition) we repeated our differential transcript usage analysis with DEXSeq2 and StageR using the same settings used in the full analysis but subsampling our four pregnant samples down to two samples (for all six possible permutations). We compared the results to the results from our full DTU analysis (using all 4 samples) in terms of log FC and the resulting set of significant genes with significant transcripts (transcript p-adj < 0.05 for at least 1 transcript per gene).

Per-transcript log FC values calculated for transcripts shared in all analyses were highly correlated for all six permutations, both when comparing pregnancy to pre-pregnancy (mean Spearman’s ρ = 0.888) and to post-parturition (mean Spearman’s ρ = 0.924) (**Table S21**). We compared the lists of genes obtained for each permutation and for the full analysis **(Fig. S5**). The DTU results were more variable than we found from the DEGs for the subsampling analysis. This is due to higher library size in PREG3 and PREG4 (**Table S1**). We used a relatively strict threshold for low-expressed genes for DTU analysis (at least 10 reads in all samples), so the addition of samples with lower overall coverage necessarily results in filtering out many low-expressed genes from the analysis (as filtering and normalisation for the DTU analysis was performed separately for each subsample).

This view is supported by principal component analysis (PCA) of transcript-level read counts after filtering and library-size normalisation (**Fig. S6**). After filtering low-expressed transcripts and normalising for library size the PCA shows clear clustering by reproductive stage, similar to **Fig. 1A**. Given these considerations, we remain confident in the robustness of our main analysis despite the differences in sample size.

**Supplementary Figures**

**Figure S1. Differential gene expression before and after pregnancy in *Z. vivipara.***

**A:** Volcano plot showing differential gene expression in the uterus post-parturition contrasted with pre-pregnancy (upregulated genes are upregulated post-parturition). Top 5 up- and downregulated genes are labelled. **B:** Over-represented Biological Process Gene Ontology (BP:GO) terms for genes upregulated post-parturition as compared to pre-pregnancy, plotted in semantic space. **C:** Over-represented BP:GO terms for genes downregulated post-parturition as compared to pre-pregnancy, plotted in semantic space.

**Figure S2. Comparison of differential gene expression (DGE) and differential transcript usage (DTU) during pregnancy in *Z. vivipara*.**

Horizontal bars (grey) show the number of significant genes for each comparison in the DGE and DTU analyses (either DEGs or genes with at least one transcript participating in DTU). Vertical bars (black) show the set of unique genes for each analysis and combination of analyses shown in the matrix below. Lizard image provided by H. Xie.

**Figure S3. Boxplots showing expression levels (normalised read counts) of two candidate genes differentially expressed during pregnancy in the *Z. vivipara* uterus.**

Plot shows normalised expression values as calculated by DESeq2.

**Figure S4. Significant DEGs when subsampling pregnant uterus samples to equalize sample size. A:** Differentially expressed genes for each permutation of n = 2 pregnant samples, and for n = 4 pregnant samples (full analysis), as compared to pre-pregnancy. Horizontal bars (grey) show the number of significant DEGs for each subsampled analysis. Vertical bars (black) show the set of unique genes for each analysis and combination of analyses shown in the matrix below. **B:** Differentially expressed genes for each permutation of n = 2 pregnant samples, and for n = 4 pregnant samples (full analysis), as compared to post-parturition. Horizontal bars (grey) show the number of significant DEGs for each subsampled. Vertical bars (black) show the set of unique genes for each analysis and combination of analyses shown in the matrix below.

**Figure S5. Genes with evidence of significant differential transcript usage when subsampling pregnant uterus samples to equalize sample size.** **A:** Differentially expressed genes for each permutation of n = 2 pregnant samples, and for n = 4 pregnant samples (full analysis), as compared to pre-pregnancy. Horizontal bars (grey) show the number of significant genes for each subsampled analysis (genes with at least one transcript participating in DTU). Vertical bars (black) show the set of unique genes for each analysis and combination of analyses shown in the matrix below. **B:** Differentially expressed genes for each permutation of n = 2 pregnant samples, and for n = 4 pregnant samples (full analysis), as compared to post-parturition. Horizontal bars (grey) show the number of significant genes for each subsampled analysis (genes with at least one transcript participating in DTU). Vertical bars (black) show the set of unique genes for each analysis and combination of analyses shown in the matrix below.

**Figure S6. Principal component analysis** **(PCA) of transcript-level read counts after filtering and normalisation.**

PCA showing transcript-level read counts for all samples after filtering out low-expressed genes with DRIMSeq and normalising for library size using DEXSeq.
